# Oligomerization-mediated autoinhibition and cofactor binding of a plant NLR

**DOI:** 10.1038/s41586-024-07668-7

**Published:** 2024-06-12

**Authors:** Shoucai Ma, Chunpeng An, Aaron W. Lawson, Yu Cao, Yue Sun, Eddie Yong Jun Tan, Jinheng Pan, Jan Jirschitzka, Florian Kümmel, Nitika Mukhi, Zhifu Han, Shan Feng, Bin Wu, Paul Schulze-Lefert, Jijie Chai

**Affiliations:** 1 School of Life Sciences, Westlake University, Institute of Biology, Westlake Institute for Advanced Study, Westlake Laboratory of Life Sciences and Biomedicine, Hangzhou, China; 2 Xianghu Laboratory, Hangzhou, China; 3 Department of Plant-Microbe Interactions, Max Planck Institute for Plant Breeding Research, Cologne, Germany; 4 School of Biological Sciences, Nanyang Technological University, Singapore, Singapore; 5 Institute of Biochemistry, University of Cologne, Cologne, Germany; 6 Cluster of Excellence on Plant Sciences, Max Planck Institute for Plant Breeding Research, Cologne, Germany

**Keywords:** Plant immunity, Plant signalling, Cryoelectron microscopy, Autoimmunity

## Abstract

Nucleotide-binding leucine-rich repeat (NLR) proteins play a pivotal role in plant immunity by recognizing pathogen effectors^1,2^. Maintaining a balanced immune response is crucial, as excessive NLR expression can lead to unintended autoimmunity^3,4^. Unlike most NLRs, the plant NLR required for cell death 2 (NRC2) belongs to a small NLR group characterized by constitutively high expression without self-activation^5^. The mechanisms underlying NRC2 autoinhibition and activation are not yet understood. Here we show that *Solanum lycopersicum* (tomato) NRC2 (*Sl*NRC2) forms dimers and tetramers and higher-order oligomers at elevated concentrations. Cryo-electron microscopy shows an inactive conformation of *Sl*NRC2 in these oligomers. Dimerization and oligomerization not only stabilize the inactive state but also sequester *Sl*NRC2 from assembling into an active form. Mutations at the dimeric or interdimeric interfaces enhance pathogen-induced cell death and immunity in *Nicotiana* *benthamiana*. The cryo-electron microscopy structures unexpectedly show inositol hexakisphosphate (IP_6_) or pentakisphosphate (IP_5_) bound to the inner surface of the C-terminal leucine-rich repeat domain of *Sl*NRC2, as confirmed by mass spectrometry. Mutations at the inositol phosphate-binding site impair inositol phosphate binding of *Sl*NRC2 and pathogen-induced *Sl*NRC2-mediated cell death in *N. benthamiana*. Our study indicates a negative regulatory mechanism of NLR activation and suggests inositol phosphates as cofactors of NRCs.

## Abstract (web summary)

Cryo-electron microscopy reveals that the tomato immune receptor NRC2 forms oligomers to stabilize its inactive state and sequester it from activation, with inositol phosphates acting as immunoregulatory cofactors.

## Main

Plants have developed a two-tiered immune system to detect and respond to potential threats in their environment^6,7^. The first line of defence, known as pattern-triggered immunity, is initiated when membrane-resident pattern recognition receptors recognize evolutionary conserved microbe-derived molecular patterns on the cell surface. However, some pathogens have evolved effector molecules that can suppress pattern-triggered immunity to facilitate infection. In response, plants have evolved resistance proteins that typically recognize strain-specific effectors, leading to a stronger defence response called effector-triggered immunity. Effector-triggered immunity often induces a hypersensitive response, a regulated host cell death restricted to sites of attempted infection.

**Fig. 1:**
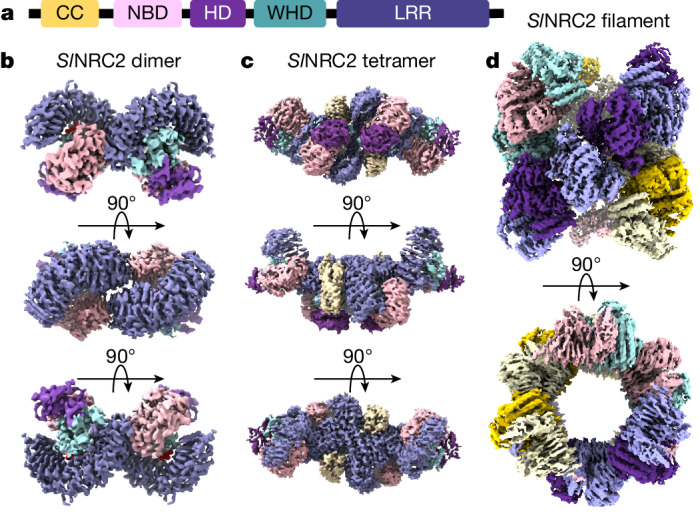
Three-dimensional reconstructions of *Sl*NRC2 dimer, tetramer and filament. **a**, Schematic diagram of domain structure of *Sl*NRC2. **b**–**d**, Different orientations of the 3D reconstructions of *Sl*NRC2 dimer (at 2.84 Å resolution) (**b**), tetramer (at 3.17 Å resolution) (**c**) and filament (at 3.6 Å resolution) (**d**). Colour codes for subdomains of *Sl*NRC2 dimer and tetramer are shown in **a** and the individual protomers in the filament are distinguished by their respective chain colours in which one coloured protomer is sandwiched between two adjacent protomers of the same colour in one strand. CC, coiled coil.

Most resistance proteins are intracellular nucleotide-binding leucine-rich repeat (NLR) receptors which play a crucial role in plant defence against pathogen invasion^1,8^. NLR receptors are multidomain proteins, which comprise a central nucleotide-binding domain (NBD) and a C-terminal leucine-rich repeat (LRR) domain (Fig. 1a). They can be categorized into different subfamilies on the basis of the presence of specific N-terminal domains such as the coiled-coil domain. In healthy plants, NLRs are bound to ADP and kept inactive through intradomain interactions^9^. Recognition of pathogen effectors triggers conformational changes in NLRs and the exchange of ADP for ATP, converting them into an active conformation. These molecular events ultimately lead to NLR oligomerization and formation of higher-order protein complexes known as resistosomes^1,7–19^. NLR resistosomes integrate signalling through calcium (Ca^2+^)-permeable channels^10–15,20,21^, triggering downstream defence mechanisms. High-level expression of many NLRs in plants results in autoactivation and hypersensitive-response cell death in the absence of pathogens^3^. This suggests that the activation of NLRs can be induced by the accumulation of NLR proteins, as evidenced by the concentration-dependent hypersensitive-response cell death observed with the *Arabidopsis thaliana* NLR RPS4 (ref. ^4^). Hence, NLR expression is tightly regulated in plants to prevent autoimmunity and ensure a balanced immune response^22^.

NLRs responsible for detecting effectors are commonly known as ‘sensor’ NLRs. These often function together with downstream signalling NLRs which translate recognition into immune activation and are referred to as ‘helper’ NLRs^23,24^. In the Solanaceae family, helper coiled-coil NLRs, specifically referred to as NLR required for cell death (NRCs), play a crucial role in enabling the function of numerous sensor NLRs^25–27^. NRCs, including NRC2, NRC3 and NRC4, belong to a small core set of NLRs that are highly expressed in the absence of pathogens compared to most other NLRs^5^. The immune response triggered by a particular sensor NLR can be dependent on one or several helper NRCs. For instance, the coat protein (CP) of potato virus X (PVX) is recognized by the LRR domain of the sensor NLR Rx^28^, presumably leading to the release of Rx NBD and subsequent activation of *Sl*NRC2 and *Sl*NRC3 (refs. ^28,29^). Once activated, NRCs form resistosomes to mediate immune signalling^17,18^. In contrast to the pentameric resistosomes of some coiled-coil NLRs^11,13–15^, the *Sl*NRC4 resistosome has recently been shown to form hexamers to trigger effector-triggered immunity^19^.

Despite significant progress in understanding NRC signalling, the structural mechanisms underlying the autoinhibition and activation of NRCs remain elusive. In this study, we discovered that *Sl*NRC2 forms dimers and tetramers and higher-order oligomers at elevated concentrations. Using cryo-electron microscopy (cryo-EM), we performed structural analyses and found that *Sl*NRC2 adopts an inactive conformation in both the dimeric and oligomeric states. Structural comparison shows that *Sl*NRC2 dimerization and oligomerization act to stabilize the inactive conformation. Impairment of *Sl*NRC2 dimerization and oligomerization enhances CP-induced cell death in *N. benthamiana*, supporting an inhibitory role of intermolecular interactions in *Sl*NRC2 activation. The cryo-EM structures unexpectedly show that an inositol phosphate (IP) molecule, primarily IP_6_ or IP_5_ (IP_6/5_), binds to the C-terminal LRR domain as confirmed by mass spectrometry (MS). The binding of IP_6/5_ to this site is required for CP-induced cell death in *N. benthamiana*, suggesting a cofactor role of IPs in modulating *Sl*NRC2 signalling. Overall, our findings provide insights into the complex negative regulation of plant NLR activation and raise questions about the role of IPs in NLR-immune signalling.

## Inactive *Sl*NRC2 forms several oligomers

**Extended Data Fig. 1:**
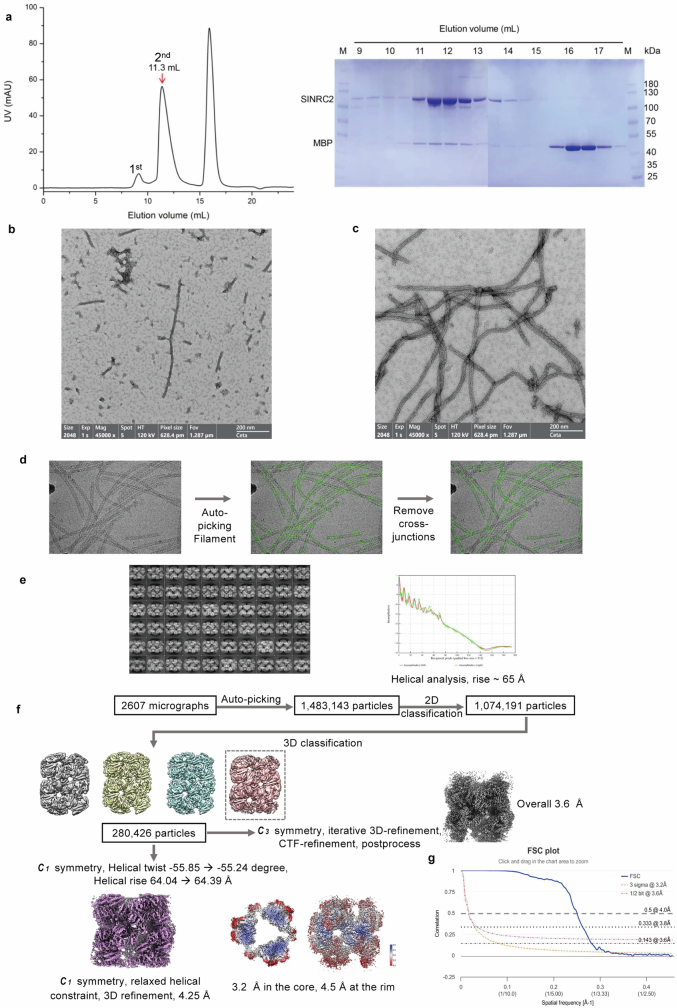
Purification of *Sl*NRC2 and flowcharts for cryo-EM structure determination of *Sl*NRC2 filaments. **a**. Purification of *Sl*NRC2 from insect cells. Maltose binding protein (MBP) fused to *Sl*NRC2 at the N-terminus was purified using affinity chromatography. The purified protein was digested with PreScission Protease to remove the N-terminal MBP tag and the protein was further purified using gel filtration. Shown on the left side is the gel filtration profile of *Sl*NRC2, the right side shows SDS-PAGE analysis of different protein fractions followed by Coomassie blue staining. Purification of the *Sl*NRC2 protein was performed more than 3 times. **b**. A representative negative staining image of the proteins from the 1^st^ peak in (a). The negative staining was performed for each protein preparation and showed similar results. **c**. A representative negative staining image of the protein from the 2^nd^ peak in (a) after concentration. The negative staining was performed for each protein preparation and showed similar results. **d**. A representative cryo-EM micrograph of *Sl*NRC2 filaments. A cryo-EM dataset comprising 2,670 images was acquired, providing sufficient information to resolve the structure of the complexes. **e**. Representative 2D averages of *Sl*NRC2 filaments. **f**. Procedure for 3D reconstruction of *Sl*NRC2 filaments. **g**. FSC curve at 0.143 of the final reconstruction of *Sl*NRC2 filament.

**Extended Data Fig. 2:**
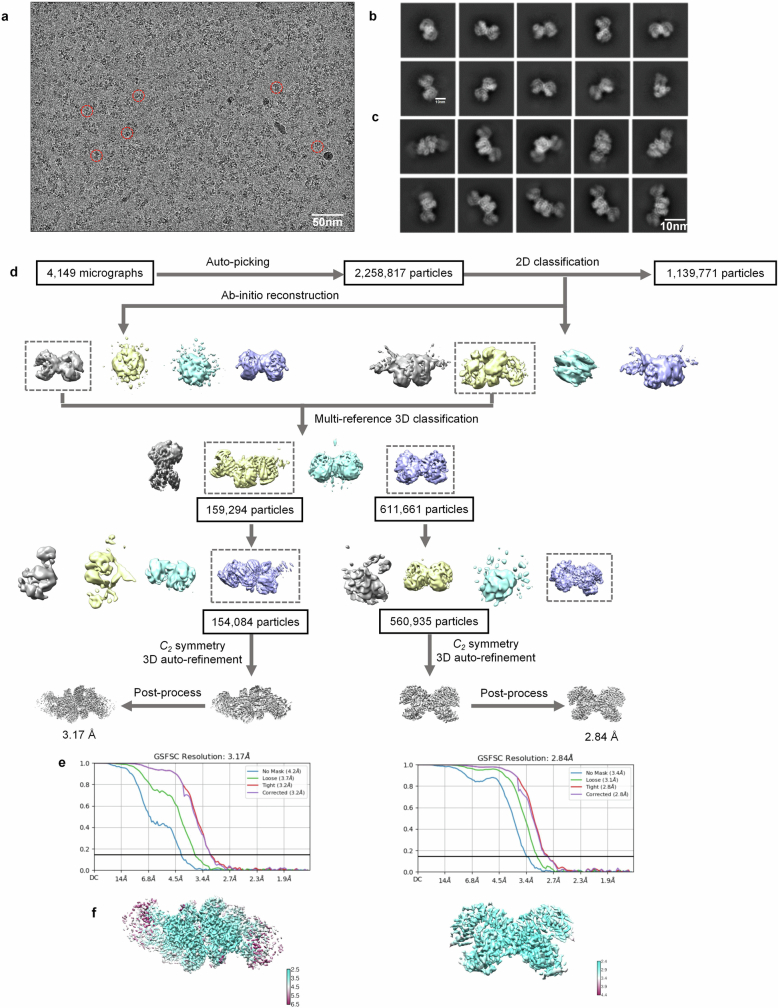
Cryo-EM reconstructions of dimeric and tetrameric *Sl*NRC2. **a**. Representative cryo-EM micrograph of the *Sl*NRC2 complexes. A cryo-EM dataset comprising 4,147 images was acquired, providing sufficient information to resolve the structure of the complexes. **b**. Representative views of 2D class averages of *Sl*NRC2 dimers. **c**. Representative views of 2D class averages of *Sl*NRC2 tetramers. **d**. The cryo-EM image processing workflow. **e**. FSC curves at 0.143 of the final reconstruction of *Sl*NRC2 dimers and tetramers. **f**. Representative views of 3D class averages of *Sl*NRC2 dimers and tetramers.

To investigate the structural mechanism of *Sl*NRC2 autoinhibition, full-length *Sl*NRC2 was expressed and purified from insect cells. Gel filtration analysis of the purified protein showed that *Sl*NRC2 eluted at a position corresponding to an apparent molecular weight of approximately 200–300 kDa (Extended Data Fig. 1a). Additionally, *Sl*NRC2 was also observed in a peak at the void volume. Electron microscopy negative-staining indicated that *Sl*NRC2 from this peak contained filaments and aggregates (Extended Data Fig. 1b). When concentrated, the *Sl*NRC2 protein from the 200–300 kDa peak also formed filaments with similar morphology to those directly eluted from the void volume (Extended Data Fig. 1c). Both the apparently dimeric protein and the filaments from the concentrated protein were subjected to cryo-EM analysis. For the apparently dimeric *Sl*NRC2 sample, a total of 1,139,771 individual particles were used for reference-free two-dimensional (2D) classification. In addition to dimers, the 2D averaging also indicated the presence of a small percentage of tetrameric *Sl*NRC2 (Extended Data Fig. 2). Three-dimensional (3D) classification was performed using the 2D averages of dimeric and tetrameric *Sl*NRC2. Further analysis was then conducted on a subset of 611,661 particles for the dimer and 159,294 particles for the tetramer, resulting in a cryo-EM density map with resolutions of 2.84 and 3.17 Å (Fig. 1b,c, Extended Data Fig. 2 and Extended Table 1), respectively. The cryo-EM structure of the filament was determined following the methods previously described^30^. A total of 280,426 particles with more coherent helical parameters were used for the final 3D refinement process (Extended Data Fig. 1d–g). The resulting cryo-EM reconstruction of the filament sample achieved an overall resolution of 3.6 Å (Fig. 1d, Extended Data Fig. 1d–g and Extended Table 1). AlphaFold2 prediction of *Sl*NRC2 was first docked to the density map of the dimeric sample and then refined using PHENIX^31^. The final refined *Sl*NRC2 dimer was docked to the cryo-EM density maps of the tetramer and filament followed by PHENIX refinement. In contrast to the tetrameric (Fig. 1c) and filamentous (Fig. 1d) *Sl*NRC2, the coiled-coil domains in the dimeric *Sl*NRC2 were not well defined (Fig. 1b) and hence were not included in the final refined model.

**Extended Data Fig. 3:**
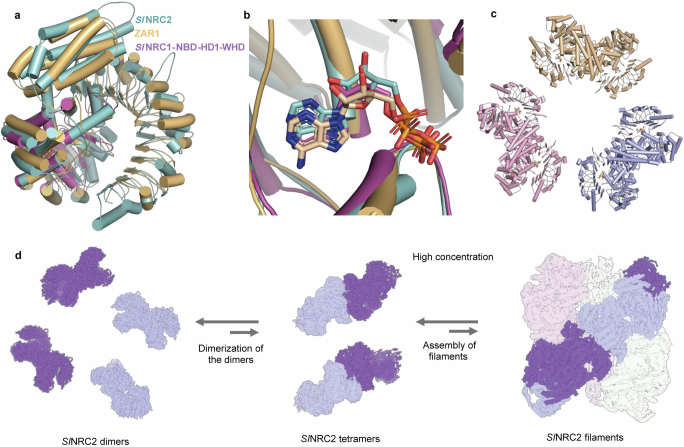
Structural analysis of inactive *Sl*NRC2 oligomeric states and a proposed working model for the assembly of *Sl*NRC2 filament. **a**. Structural alignment of *Sl*NRC2 (from the dimer), inactive ZAR1 (PDB code: 6J5W) and inactive *Sl*NRC1 NBD-HD1-WHD (PDB code: 6S2P). **b**. A zoomed in view of the structural alignment in (**a**) around the ADP-binding site. **c**. Top view of the structure of *Sl*NRC2 filaments. **d**. Under non-challenged conditions, *Sl*NRC2 protein exists in a dimer-tetramer equilibrium in solution. The homodimerization of the *Sl*NRC2 dimer is through a “back-to-back” interaction. At elevated concentrations, tetrameric *Sl*NRC2 can interact with dimeric *Sl*NRC2, resulting in propagation of the tetramer and formation of higher-order *Sl*NRC2 oligomers. The *Sl*NRC2 oligomers may interact with each other to form triple helical filaments.

Structural similarity analysis using Foldseek^32^ showed that the cryo-EM structure of *Sl*NRC2 from the dimer, tetramer and filaments resembles that of inactive ZAR1 (Extended Data Fig. 3a). The cryo-EM structure of *Sl*NRC2 NBD–helix domain 1 (HD1)–winged-helix domain (WHD) can also be well aligned with the crystal structure of *Sl*NRC1 NBD–HD1–WHD^33^ (Extended Data Fig. 3a). These structural observations indicate that *Sl*NRC2 in the cryo-EM structures is in an autoinhibited state. Consistently, binding of an ADP molecule was observed between the NBD and HD1 of *Sl*NRC2 (Extended Data Fig. 3b).

## Structural mechanisms of *Sl*NRC2 dimerization and oligomerization

**Fig. 2:**
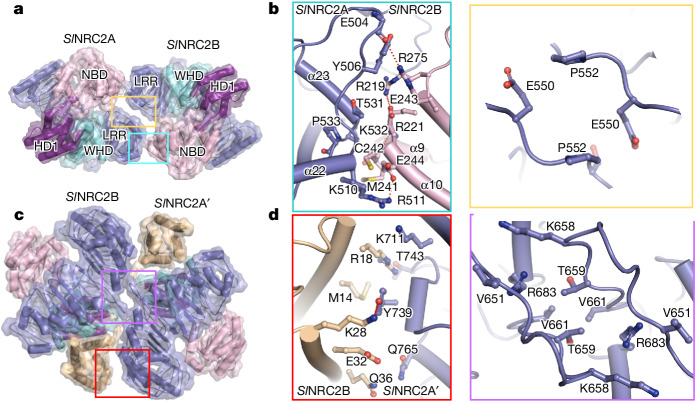
Structural mechanisms of *Sl*NRC2 oligomerization. **a**,**c**, *Sl*NRC2 ‘head-to-head’ dimer (**a**) and ‘back-to-back’ dimer within the tetramer (**c**) shown in transparent surface and cartoon. Colours codes for subdomains of *Sl*NRC2 are the same as in Fig. 1a. **b**,**d**, Detailed interactions highlighted in **a** (**b**) and in **c** (**d**). Red dashed lines represent polar interactions.

The *C*_2_ symmetry-related *Sl*NRC2 molecules in the dimer form a ‘head-to-head’ interaction through two interfaces (Fig. 2a). The first interface is facilitated by the packing of the N-terminal outer surface of the LRR domain from one protomer (*Sl*NRC2A) against one end of the three-helix bundle of the NBD domain from the other protomer (*Sl*NRC2B) (Fig. 2b, left panel). This interface involves tight contacts of the C-terminal end of the α-helix from the second LRR of *Sl*NRC2A with a short loop region which links α9 and α10 in *Sl*NRC2B (Fig. 2b, left panel). The side chains of *Sl*NRC2A Lys532 and *Sl*NRC2B Arg221 interact with *Sl*NRC2B α9 and *Sl*NRC2A α23, respectively. Additionally, *Sl*NRC2A Tyr506 from the loop region N-terminal to α22 interacts with *Sl*NRC2B Glu271 and Arg275. The N-terminal end of the LRR domain of *Sl*NRC2A symmetrically stacks with its equivalent from the *Sl*NRC2B molecule (Fig. 2b, right panel), forming the second interface which mediates *Sl*NRC2 dimerization.

In the cryo-EM structure of tetrameric *Sl*NRC2, the interaction between two dimers (*Sl*NRC2A–*Sl*NRC2B and *Sl*NRC2A′–*Sl*NRC2B′) is mediated through ‘back-to-back’ contact between *Sl*NRC2B and *Sl*NRC2A′ (Fig. 2c). One lateral side of the LRR domain of *Sl*NRC2A′ contacts the coiled-coil domain of *Sl*NRC2B. This interaction can serve to stabilize the coiled-coil domain which is not well defined in the dimeric *Sl*NRC2 (Fig. 1b). *Sl*NRC2A′ Tyr739 is in packing contact with the side chains of *Sl*NRC2B Met14, Arg18 and Glu32 (Fig. 2d, left panel). The carbonyl oxygen of *Sl*NRC2A′ Thr743 hydrogen bonds to *Sl*NRC2B Arg18. The symmetrical packing of the LRR domains of *Sl*NRC2B and *Sl*NRC2A′ further enhances the interaction of the two *Sl*NRC2 dimers. The stacking of two loop-out regions dominantly contributes to the symmetrical interaction around this interface (Fig. 2c and right panel of Fig. 2d).

The *Sl*NRC2 filament consists of three protofilaments. Only marginal contacts mediate the interprotofilament interactions (Extended Data Fig. 3c). Both dimeric and tetrameric *Sl*NRC2 structures are present in the filament. Each protofilament contains several copies of tetrameric *Sl*NRC2. Filament propagation probably involves the incorporation of dimeric *Sl*NRC2 into pre-existing filaments (Extended Data Fig. 3d).

## Oligomerization enhances autoinhibition of *Sl*NRC2

**Fig. 3:**
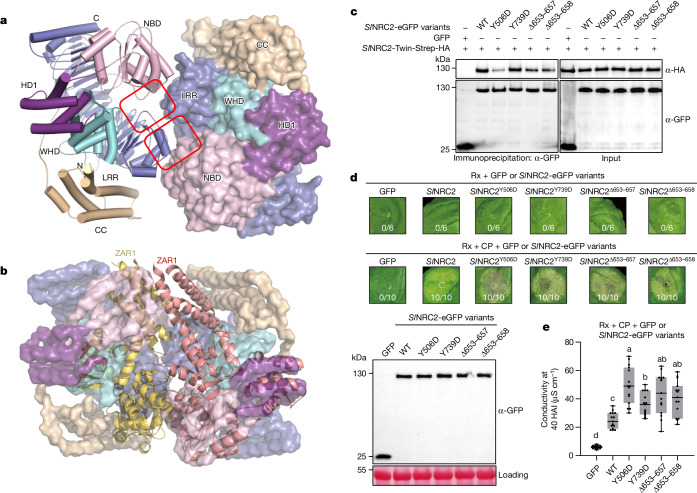
Oligomer-disrupting mutations of *Sl*NRC2 promote CP-induced cell death in *N. benthamiana.* **a**, *Sl*NRC2 dimerization stabilizes its inactive conformation. Dimeric *Sl*NRC2 (from the *Sl*NRC2 tetramer) with the right subunit shown in a surface and the left in a cartoon representation. Subdomains are indicated. ‘N’ and ‘C’ represent the N and C terminus, respectively. **b**, Structural alignment of dimeric *Sl*NRC2 (in surface representation) and a lateral dimer of the ZAR1 resistosome (in cartoon representation). The NBD–HD segment in the right subunit of *Sl*NRC2 was used as the template to align with a lateral dimer of the ZAR1 resistosome (PDB code 6J5T). For clarity, the C-terminal LRR domain and the N-terminal α1 helix are not shown. **c**, Co-immunoprecipitation assay for testing self-association of *Sl*NRC2 oligomer-disrupting mutations. The *nrc2/3/4* mutant leaves co-expressing *SlNRC2-Twin-Strep-HA* and the indicated *SlNRC2-eGFP* variants were subjected to immunoprecipitation with anti-GFP, followed by immunoblotting with indicated antibodies. Two independent experiments were performed with similar results. **d**, Cell death phenotypes mediated by *Sl*NRC2 oligomer-disrupting mutations. *SlNRC2-eGFP* variants, *Rx-HA-StrepII* were co-expressed with or without *CP-FLAG* in *nrc2/3/4* mutant leaves. The representative figure is shown from six or ten replicates, respectively. The protein levels of the *Sl*NRC2 variants are shown at the bottom. Ponceau S staining of RuBisCO was used as a loading control. **e**, Ion leakage assay of *Sl*NRC2 oligomer-disrupting mutations in Rx-triggered cell death. The assay was performed as described in **d** at 40 h after agro-infiltration (HAI). Results from three independent experiments (*n* = 15, five biological independent samples for each experiment); different letters indicate significant differences (analysed by one-way analysis of variance (ANOVA) with Tukey’s multiple comparisons test, adjusted *P* < 0.05). In box and whiskers, the centre line indicates the median, the bounds of the box show the 25th and 75th percentiles and whiskers represent the minimum to maximum values. WT, wild type. Source Data

Structural studies have shown that NLR activation involves notable structural reorganization between the N-terminal NBD–HD1 and the C-terminal WHD–LRR segments^1,7^. In addition, the coiled-coil domain also undergoes fold remodelling during NLR activation. In the structure of the dimeric *Sl*NRC2, the N-terminal end of the *Sl*NRC2A LRR domain is wedged between the NBD and LRR domains of *Sl*NRC2B (Fig. 3a), hence stabilizing the relative conformation of these two structural domains. NLR oligomerization during activation follows a conserved domain organization^1^. In this mechanism, the NBD from one NLR protomer aligns with the opposite side of the NBD from the other NLR protomer to form a lateral dimer. This stacking of NBDs on adjacent sides ensures the structural integrity of the oligomeric NLR complex. A structural comparison between the inactive *Sl*NRC2 dimer and a lateral dimer from the ZAR1 resistosome showed that the *Sl*NRC2 dimerization interfaces overlap with the lateral dimeric ZAR1 surface mediating resistosome assembly (Fig. 3b). The *Sl*NRC2 dimerization can be further stabilized through the formation of tetramer and filaments. These structural findings suggest that *Sl*NRC2 dimerization can serve to inhibit the activation of the NLR protein, with further reinforcement by the formation of higher-order oligomers.

**Extended Data Fig. 4:**
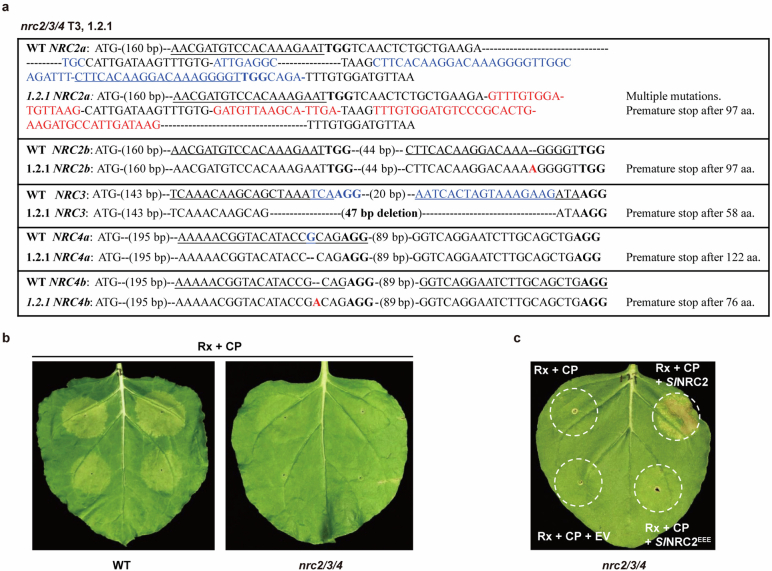
Genotypes and phenotypes of the *N. benthamiana nrc2/3/4* mutant. **a**. Amplicon sequencing results of *NRC* loci of the *nrc2/3/4* mutant line 1.2.1. Sequences of the guide RNAs target *NRC2*, *NRC3* and *NRC4* are marked by underlining. The mutation sites are highlighted in red in the mutants and the different base pairs of the WT genes and mutants are highlighted in blue. **b**. Rx-mediated cell death is compromised in the *nrc2/3/4* mutant line. *Rx-HA-StrepII* and *CP-FLAG* were transiently expressed in leaves of WT *N. benthamiana* and *nrc2/3/4* mutant line 1.2.1. The pictures were taken 3 DAI. **c**. Reconstitution of the Rx-NRC signaling complex in *nrc2/3/4* mutant line. *Rx-HA-StrepII*, *CP-FLAG, SlNRC2* or *SlNRC2*^*EEE*^*-eGFP* (L9E/L13E/L17E) were co-expressed as indicated in the *nrc2/3/4* line 1.2.1. The pictures were taken 3 DAI.

To test this model, we first generated *N. benthamiana* plants harbouring mutations in *NbNRC2*, *NbNRC3* and *NbNRC4* by gene editing and selected triple mutant plants lacking all three helper NLRs (*nrc2/3/4*) (Extended Data Fig. 4a,b,c). Using *Agrobacterium tumefaciens*-mediated transient gene expression in leaves of *N. benthamiana nrc2/3/4* plants, we then co-expressed *SlNRC2-Twin-Strep-HA* with different *SlNRC2-eGFP* constructs to investigate their interaction (Fig. 3c). Immunoprecipitation assays showed an interaction between the two differentially tagged *Sl*NRC2 proteins (Fig. 3c), supporting the biochemical and structural data concerning *Sl*NRC2 self-association (Fig. 1b,c,d and Extended Data Fig. 1a). The self-association was substantially impaired by the *Sl*NRC2^Y506D^ mutation (Fig. 3c), which is predicted to disrupt the first *Sl*NRC2 dimerization interface (Fig. 2b, left panel). Mutations at the dimer–dimer interface (*Sl*NRC2^∆653–657^ and *Sl*NRC2^∆653–658^) (Fig. 2d, right panel) exhibited a similar but less pronounced effect on *Sl*NRC2 self-association. By comparison, *Sl*NRC2^Y739D^ from the same interface had minimal impact on *Sl*NRC2 self-association (Fig. 2d, left panel).

Then we assessed how these *Sl*NRC2 mutations affected CP-induced *Sl*NRC2-mediated cell death in *nrc2/3/4 N. benthamiana*. To this end, we individually co-expressed these *SlNRC2* mutants with *CP* and *Rx* in the *nrc*2*/3/4* mutant. As expected, co-expression of wild-type *SlNRC2* with *Rx* and *CP* resulted in hypersensitive-response cell death in the mutant plants (Fig. 3d). Although co-expression of various *SlNRC2* mutants with *Rx*, but without *CP*, did not yield any discernible phenotype in *nrc*2*/3/4* plants, their co-expression with *CP* and *Rx* significantly enhanced hypersensitive-response cell death compared to wild-type *SlNRC2*, albeit to varying degrees (Fig. 3d). Notably, although *Sl*NRC2^Y739D^ had little impact on *Sl*NRC2 self-association, the mutation significantly promoted CP-induced cell death in *nrc*2*/3/4 N. benthamiana*. One reason for this could be the different sensitivity of these two assays to the mutation. Western blot analysis confirmed comparable expression levels of these *Sl*NRC2 proteins. Collectively, these findings demonstrate negative regulation of *Sl*NRC2 activation by dimerization and oligomerization. In strong support of this conclusion, quantification of CP-induced cell death through ion leakage assays demonstrated higher conductivity of these *Sl*NRC2 mutants than wild-type *Sl*NRC2 when co-expressed with Rx and CP in *nrc2/3/4* plants (Fig. 3e).

**Extended Data Fig. 5:**
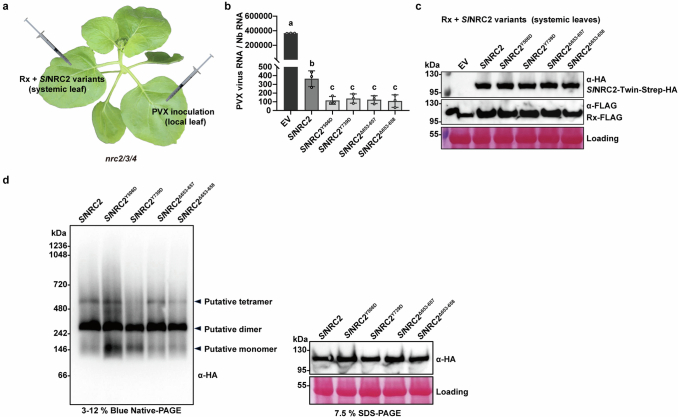
*Sl*NRC2 oligomer-disrupting mutants enhanced Rx-mediated inhibition of Potato Virus X (PVX) proliferation. **a**. Experimental set-up of PVX infection and resistance assay. **b**. qRT-PCR assay to quantify PVX virus load. RNA of *PVX coat protein (CP)* was tested and normalized to the *F-box* gene in *N. benthamiana*. PVX was inoculated by agroinfiltration at the lower leaf (local leaf) at OD_600_ = 0.005, *Rx-FLAG* and *SlNRC2-Twin-Strep-HA* variants were co-expressed at the upper leaf (systemic leaf) with OD_600_ = 0.2. Empty vector (EV) was used as a negative control for *SlNRC2* and its variants. At 7 DAI, the systemic leaves were used for testing PVX virus load. Data are presented as mean ± s.d. for *n* = 3 independent experiments, with individual data points overlaid. Different letters indicate significant differences (*p* < *0.05*; two tailed *t*-test). **c**. Protein expression of Rx and *Sl*NRC2 or *Sl*NRC2 oligomer-disrupting mutants in **b**. were tested using SDS-PAGE and western blot with the indicated antibodies. Ponceau S staining of RuBisCO was used as a loading control. **d**. Blue Native-PAGE assay testing the oligomeric status of *Sl*NRC2 and its variants. Indicated versions of *SlNRC2-Twin-Strep-HA* were expressed in the leaves of *nrc2/3/4* mutant *N. benthamiana* plants. Protein samples were loaded onto a blue native-PAGE gel (Left panel). Putative monomers, dimers and tetramers of *Sl*NRC2 were indicated with arrows. Additionally, SDS-boiled protein samples were loaded onto an SDS-PAGE gel (Right panel) to serve as a control for the actual size of *SlNRC2*-Twin-Strep-HA. For Ponceau S staining, the same samples were run in a parallel gel. Ponceau S staining of RuBisCO was used as a loading control. Three independent experiments were performed with similar results. Source Data

Next, we infiltrated PVX virus in local leaves of *N. benthamiana* and co-infiltrated *Agrobacterium* containing *Rx-FLAG* with constructs of *SlNRC2-Twin-Strep-HA* variants encoding oligomer-disrupting mutations of *Sl*NRC2 in the upper systemic leaves (Extended Data Fig. 5a). These assays showed significantly enhanced Rx-mediated inhibition of PVX proliferation of the tested *Sl*NRC2 mutants compared to wild-type *Sl*NRC2 (Extended Data Fig. 5b,c), which indicates that *Sl*NRC2 oligomer-disrupting mutants also enhance Rx-triggered disease resistance to PVX infection. Finally, we performed Blue Native–PAGE assays of wild-type *Sl*NRC2 protein expressed in *nrc2/3/4* *N. benthamiana*, providing evidence for dimers and tetramers in planta (Extended Data Fig. 5d). In support of the functional data, oligomer-disrupting mutations of *Sl*NRC2^Y506D^ and *Sl*NRC2^Y739D^ enhanced the production of monomeric *Sl*NRC2 protein, whereas the other two tested mutations had a less noticeable effect on the oligomerization states of *Sl*NRC2 (Extended Data Fig. 5d).

## Binding of IPs in *Sl*NRC2

**Fig. 4:**
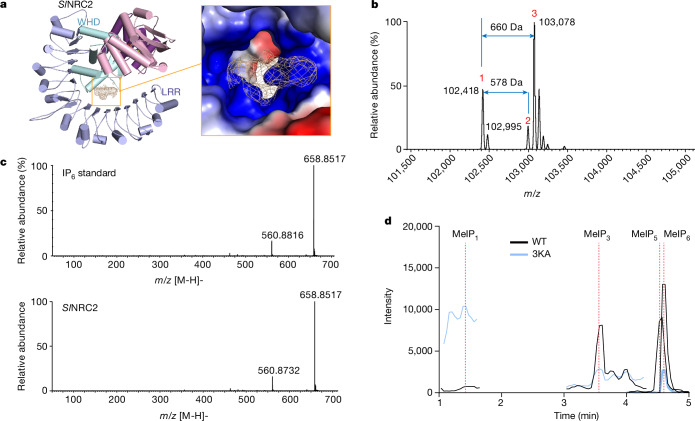
Inositol binding of *Sl*NRC2. **a**, Extra cryo-EM density between the LRR and WHD domains of dimeric *Sl*NRC2 in a highly positively charged pocket. Left panel, the difference in cryo-EM density after subtracting the *Sl*NRC2 dimer and ADP density from the reconstruction density of dimeric *Sl*NRC2. For clarity, only one *Sl*NRC2 subunit is shown. Right panel, electrostatic surface around the density difference in the left panel. **b**, Native MS analysis of *Sl*NRC2 protein purified from insect cells. Shown in the figure is a deconvoluted native mass spectrum (*m*/*z* range 101,500–103,350) of the analysis. Peak 1 (*m*/*z* = 102,418) corresponds to the mass of *Sl*NRC2 + ADP ± 5 Da, peak 2 (*m*/*z* = 102,995) to the mass of *Sl*NRC2 + ADP + IP_5_ ± 7 Da and peak 3 (*m*/*z* = 103,078) to the mass of *Sl*NRC2 + ADP + IP_6_ ± 5 Da. **c**, HRMS analysis in the negative mode of IP_6_ standard (top) and small molecules extracted from insect cell-purified *Sl*NRC2 protein (bottom). *Sl*NRC2 from **b** was denatured and hydrophilic small molecules bound to the protein were extracted. The MS/MS spectra were acquired following collision-induced dissociation fragmentation at 20 eV of the ion at *m*/*z* = 658.8517 (*z* = 1^−^). **d**, MRM-MS analysis of small molecules extracted from plant-purified *Sl*NRC2 protein. Twin-Strep-tagged *Sl*NRC2 protein was purified from *N. benthamiana* and *Sl*NRC2-bound small molecules were extracted and methylated. The methylated products were analysed by a triple Q mass spectrometer. Coloured lines represent the extract fragmentated ion chromatogram of the target analytes. Black and blue lines indicate WT and the 3KA mutant, respectively. Dashed lines indicate the analyte peaks that were integrated. From left to right MeIP_1_, MeIP_3_, MeIP_4_ and MeIP_6_.

**Extended Data Fig. 6:**
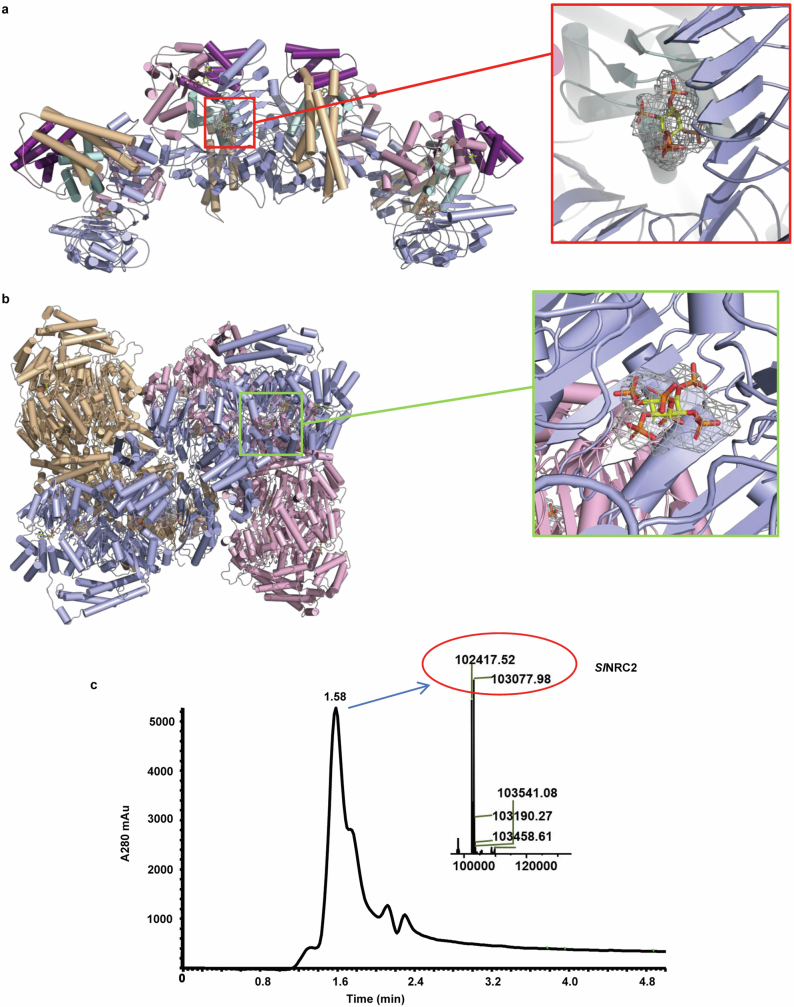
Identification of extra cryo-EM density between the WHD and LRR domains in *Sl*NRC2 tetramers, filaments and further analysis by native MS. **a**. Shown is the cryo-EM density after subtracting the tetrameric *Sl*NRC2 and bound ADP molecule density from the reconstruction map of tetrameric *Sl*NRC2. **b**. Shown is the cryo-EM density after subtracting the *Sl*NRC2 filament (12 *Sl*NRC2 subunits) and bound ADP molecule density from the reconstruction map of *Sl*NRC2 filaments. **c**. The size exclusion chromatography (SEC) profile of the purified *Sl*NRC2 protein in native mass spectrometry analysis. The deconvoluted mass from the highest peak of SEC, which corresponds to the molecular weight of the monomeric SlNRC2, is labeled (inset).

In the cryo-EM density maps of the *Sl*NRC2 dimer, tetramer and filaments, an extra round and flat-shaped density with a strong signal-to-noise ratio is observed between the WHD and LRR domains (Fig. 4a and Extended Data Fig. 6a,b). However, it does not correspond to any of the *Sl*NRC2 residues, indicating the potential binding of unknown molecule(s) at this site. Examination of the surrounding environment provides insights into the potential identities of the molecules. The density is in close proximity to seven positively charged residues, including six Lys and one Arg and several polar residues. These residues collectively create a positively charged pocket (Fig. 4a, right panel), suggesting that the unknown molecules binding to this pocket may carry several negative charges. Considering the shape of the density, we hypothesized that highly anionic IPs such as hexakisphosphate (IP_6_) or other IPs could be candidate molecules.

**Extended Data Fig. 7:**
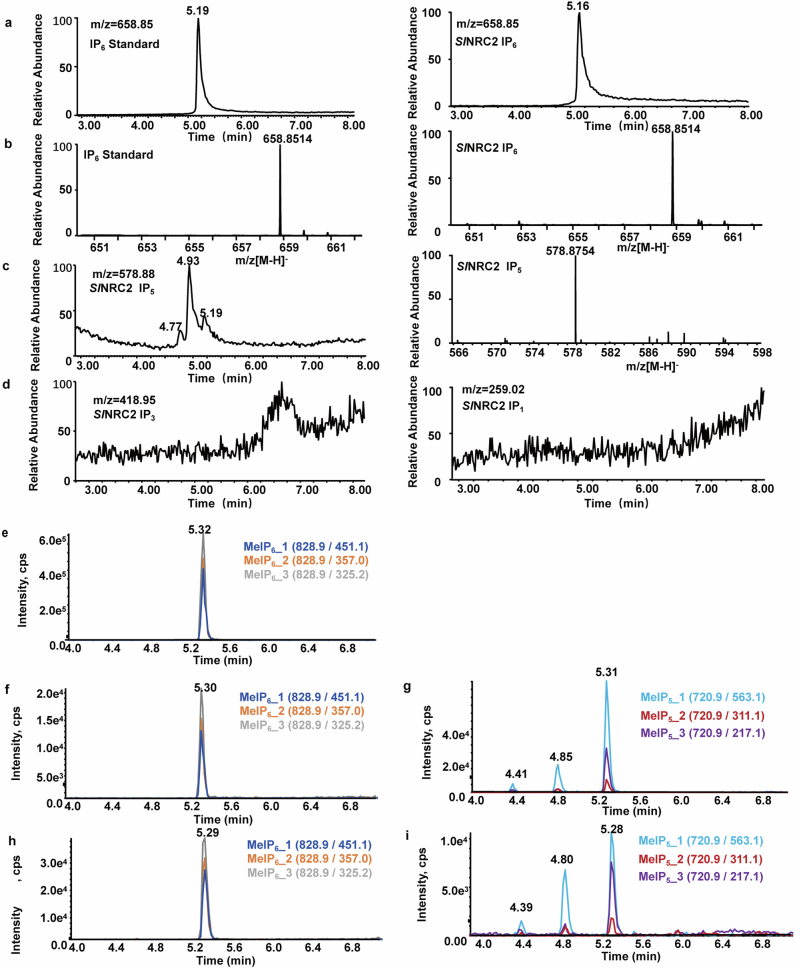
LS/LC-MS analysis of IPs bound in insect cell- and plant-purified *Sl*NRC2. **a**. Chromatograms of IP_6_ standard with retention time of 5.19 min (left) and the compound extracted from insect cell-purified *Sl*NRC2 with retention time of 5.16 (right). **b**. MS analysis of IP_6_ standard with retention time of 5.19 min (left) and the compound extracted from insect cell-purified *Sl*NRC2 with retention time of 5.16 (right). **c**. Chromatogram of IP_5_ extracted from insect cell-purified *Sl*NRC2 with retention time of 4.93 (left) and mass spectrum of the compound (right). **d**. Chromatograms of IP_3_ (left) and IP_1_ (right) extracted from insect cell-purified *Sl*NRC2. **e**-**i**. LC-MS analysis of IPs in insect cell- and plant-purified *Sl*NRC2. IP_6_ standard, small molecules extracted from insect cell-purified and *N. benthamiana*-purified *Sl*NRC2 were methylated as described in the Methods section. The methylated products were then analyzed by LC-MS in the multiple reaction monitoring (MRM) mode. Shown are representative chromatograms of the MRM analyses for methylated IP_6_ standard with retention time of 5.32 min (**e**), the methylated small molecule extracted from insect cell-purified *Sl*NRC2 with retention time of 5.30 min (**f**) and that from *N. benthamiana*-purified *Sl*NRC2 with retention time of 5.29 min (**h**), respectively. LC-MRM analysis was also performed on the methylated small molecule extracted from insect cell-purified *Sl*NRC2 (**g**) and *N. benthamiana* (**i**) to identify MeIP_5_.

To test this hypothesis, we assayed the insect cell-purified *Sl*NRC2 protein using native MS. Interestingly, the protein (at concentration of 4 μM) was monomeric under the native MS compatible conditions (0.1 M ammonium acetate and pH 6.8) (Extended Data Fig. 6c). Nonetheless, the data from the assay showed that the mass of the purified protein is compatible with that of NRC2 + IP_6_ or NRC2 + IP_5_, supporting the presence of IP_6_ and IP_5_ in the *Sl*NRC2 sample (Fig. 4b). To further confirm these results, we denatured the *Sl*NRC2 protein and extracted the presumed hydrophilic small molecules bound to *Sl*NRC2, using the previously described procedure^34^. The extractant was then subjected to liquid chromatography high-resolution MS (LC–HRMS) analysis. The LC–HRMS assay in negative mode identified an ion feature with *m*/*z* 658.8517 (*z* = 1^−^), indicating the presence of IP_6_ (theoretical molecular mass 659.8614, 3.3 ppm mass shift) (Fig. 4c). The retention time and molecular weight of this molecule closely matched those of the IP_6_ standard (Extended Data Fig. 7a,b,e–i). Additionally, the assays detected the presence of pentakisphosphate (IP_5_) in the *Sl*NRC2 sample (Extended Data Fig. 7c,e–i). By comparison, IP_1_ and IP_3_ were not strongly detected in the *Sl*NRC2 sample (Extended Data Fig. 7d). Together, this shows that insect cell-purified *Sl*NRC2 primarily binds to IP_6_ and IP_5_.

To investigate whether *Sl*NRC2 binds IPs in planta, we purified an N-terminally Twin-Strep-tagged *Sl*NRC2 from wild-type *N. benthamiana*. The plant-purified *Sl*NRC2 protein was denatured and the IPs bound to the protein were extracted and methylated using a previously described method^35^. The methylated IP (MeIPs) products were then analysed using LC–MS in the multiple reaction monitoring (MRM) mode. The LC–MS assay clearly detected the presence of MeIP_6_ and MeIP_5_ in the plant-derived *Sl*NRC2 protein (Fig. 4d and Extended Data Table 2). Additionally, MeIP_3_ with a comparable level to MeIP_5_ was also found in the sample. In summary, our data indicate that *Sl*NRC2 has significant binding affinity for IP_5/6_ both in the protein purified from insect and plant cells and for IP_3_ in the protein purified from *N. benthamiana*. This conclusion is further reinforced by a recent cryo-EM investigation which examined *Nb*NRC2 isolated from *N. benthamiana* and seemed to show the presence of additional cryo-EM density between the WHD and LRR domains^36^.

## IP binding is required for *Sl*NRC2-mediated hypersensitive-response cell death in *N. benthamiana*

**Fig. 5:**
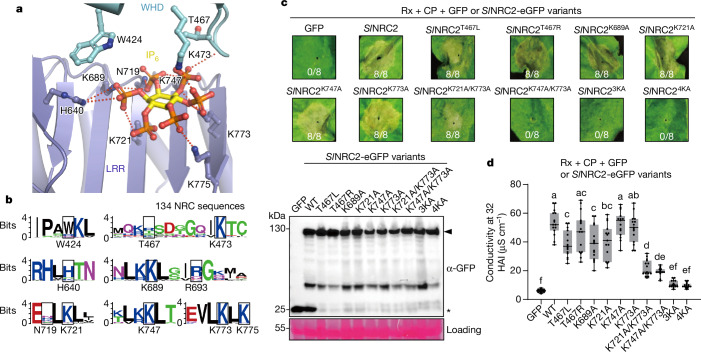
Inositol binding is required for *Sl*NRC2-triggered cell death in *N. benthamiana.* **a**, Specific recognition of IP_6_ by *Sl*NRC2. Salt bridges are shown as dashed lines. **b**, IP-binding sites of *Sl*NRC2 are conserved among NRCs from different plant species. Shown are sequence motif logos which illustrate the relative frequency of IP-binding residues and information content of the position (bits, *y* axis) across 134 NRC sequences. IP-binding sites are highlighted with a box. The logos were created by WebLogo. **c**, Hypersensitive-response phenotypes of *Sl*NRC2 and *Sl*NRC2 IP-binding mutants in Rx-triggered cell death. Co-expression of *SlNRC2*-*eGFP* variants, *Rx-HA-StrepII* and *CP-FLAG* in *N. benthamiana nrc2/3/4* mutant leaves. The representative figure is shown from at least eight replicates. *Sl*NRC2^3KA^ is short for *Sl*NRC2^K721A/K747A/K773A^, *Sl*NRC2^4KA^ is short for *Sl*NRC2^K689A/K721A/K747A/K773A^. Lower bottom panel, protein expression of the WT and mutants of *Sl*NRC2 in the *N. benthamiana* leaves were tested using SDS–polyacrylamide gel electrophoresis (SDS–PAGE) and subsequent immunoblotting with anti-GFP. Ponceau S staining of RuBisCO was used as a loading control. Arrow and asterisk indicate the positions of *Sl*NRC2-eGFP variants and GFP protein, respectively. **d**, Ion leakage assay of *Sl*NRC2 variants of IP_6/5_ binding sites in *nrc2/3/4* mutant *N. benthamiana* leaves. The assay was performed as described in **c** after agro-infiltration of *Rx-HA-StrepII, CP-FLAG* and *SlNRC2-eGFP* variants for 32 h. Results from three independent experiments (*n* = 15, five biological independent samples for each experiment), Different letters indicate significant differences (analysed by one-way ANOVA with Tukey’s multiple comparisons test, adjusted *P* < 0.05). In box and whiskers, the centre line indicates the median, the bounds of the box show the 25th and 75th percentiles, the whiskers indicate minimum to maximum values. Source Data

In the cryo-EM structures, IP_6/5/3_ interacts with particular residues from the WHD and LRR domains of *Sl*NRC2 (Fig. 5a). As IP_6_ was most prominently detected, this IP molecule was modelled in the extra cryo-EM density in the dimeric *Sl*NRC2 (Fig. 5a). The modelled IP_6_ contains five equatorial phosphate groups and one less-well defined axial phosphate group. As anticipated, IP_6_ interaction with *Sl*NRC2 is predominantly mediated by salt bridges. Among the interacting residues, all seven IP_6_-interacting residues from the LRR domain, except for His640 and Lys689, primarily support the bound IP_6_ through polar interactions. On the other hand, these two LRR residues, along with the three residues from the WHD, directly interact with IP_6/5_ from the top (Fig. 5a). These interactions result in a total of 13 salt bridges and 4 hydrogen bonds between IP_6_ and *Sl*NRC2. The axial phosphate group in the modelled IP6 establishes no hydrogen bond with *Sl*NRC2. Sequence alignment showed that the IP-binding site of *Sl*NRC2 exhibits high conservation in NRCs from different solanaceous plant species (Fig. 5b), suggesting a conserved IP-binding activity among this family of NLRs.

To assess the significance of the IP-binding activity of *Sl*NRC2 in CP-induced immune signalling, we engineered mutations (*Sl*NRC2^K721A/K747A/K773A^ called *Sl*NRC2^3KA^, *Sl*NRC2^K721A/773A^ and *Sl*NRC2^K747A/773A^) in the IP-binding residues and subsequently purified the resulting mutant from wild-type *N. benthamiana*. Using the MS assay described previously, we first investigated the impact of these mutations on the IP-binding activity of *Sl*NRC2. In further support of our biochemical and structural observations, the data from the assay showed that 3KA mutations significantly disrupted the binding of IPs (IP_3,5,6_) to *Sl*NRC2 in *N. benthamiana* (Fig. 4d). Although single mutations of the IP-interacting residues had no detectable effect on CP-induced hypersensitive-response cell death in *nrc2/3/4 N. benthamiana*, simultaneous mutations of two IP-interacting residues *Sl*NRC2^K721A/773A^ or *Sl*NRC2^K747A/773A^ strikingly reduced the cell death phenotype. Supporting a critical role of IP binding in *Sl*NRC2 signalling, the 3KA mutation resulted in loss of CP-induced hypersensitive-response cell death (Fig. 5c). As anticipated, a similar loss of CP-induced hypersensitive-response cell death was also observed with *Sl*NRC2^K689A/K721A/K747A/K773A^ (*Sl*NRC2^4KA^) (Fig. 5c). In further support of these data, ion leakage assays showed that the IP-binding mutations *Sl*NRC2^K747A/773A^, *Sl*NRC2^3KA^ and *Sl*NRC2^4KA^ greatly impaired or nearly abolished the conductivity induced by CP in *nrc2/3/4 N. benthamiana* (Fig. 5d). Collectively, these functional results in planta, supported by biochemical data, provide evidence for the essential role of IPs in *Sl*NRC2-mediated immune signalling.

## Discussion

**Extended Data Fig. 8:**
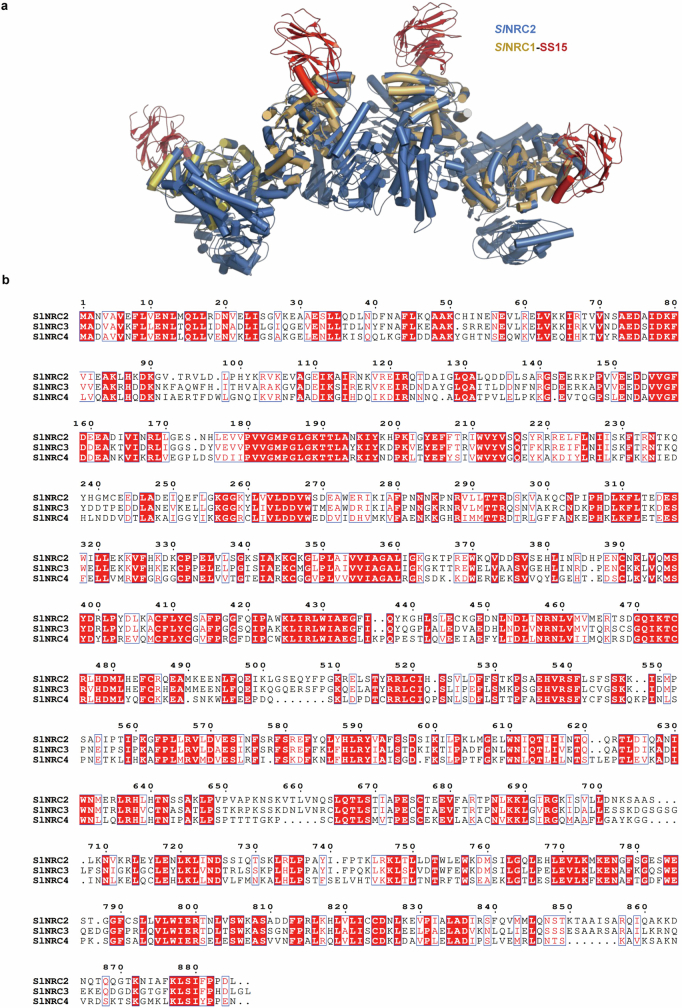
The inhibitory oligomerization of *Sl*NRC2 is not interfered by the pathogen effector SS15 and the residues surrounding its oligomerization surface are not conserved in *Sl*NRC3 and *Sl*NRC4. **a**. Structural alignment of the *Sl*NRC2 tetramer with the crystal structure of the *Sl*NRC1-SS15 complex (PDB code: 8BV0). **b**. The protein sequence alignment of *Sl*NRC2, *Sl*NRC3 and *Sl*NRC4 with residues surrounding the oligomerization surfaces of *Sl*NRC2 labeled with dark stars.

Previous structural investigations have shown a conserved autoinhibition mechanism mediated by intradomain interactions of NLR proteins^9^. This mechanism is further supported by the current cryo-EM structures of *Sl*NRC2. However, in contrast to the monomeric structures of other autoinhibited NLRs, our findings show that inactive *Sl*NRC2 is likely to form dimer-based oligomers, including dimers in solution. Self-association of *Sl*NRC2 was also demonstrated in planta in our present study and a recent publication^36^. Consistent with these findings, overexpression of *Sl*NRC2, but not *Sl*NRC4, resulted in the formation of filament-like structures inside plant cells^37^. *Sl*NRC2 dimerization and oligomerization not only stabilize the inactive conformation but also directly interfere with the assembly of the *Sl*NRC2 resistosome. In addition, *Sl*NRC2 oligomerization may lead to sequestration of the signalling coiled-coil domain, which is disordered in dimeric *Sl*NRC2. Therefore, this type of oligomerization is referred to as inhibitory oligomerization to distinguish it from the oligomerization involved in resistosome assembly. Plant pathogen effectors have evolved to exploit intermolecular interactions to suppress *Sl*NRC signalling. Specifically, a virulence effector from cyst nematodes binds to a surface area on the NBD–HD1 segment of *Sl*NRC2 (ref. ^38^), which is predicted to be involved in the assembly of the *Sl*NRC2 resistosome. By binding to this region, the effector can directly inhibit the assembly of the *Sl*NRC2 resistosomes, although the effector binding area is different from the inhibitory oligomerization surfaces (Extended Data Fig. 8a). Binding of the effector also acts to prevent intramolecular rearrangement of NBD and HD1 and consequently block *Sl*NRC2 activation^38^.

Animal NLR NLRP3 has also been found to form oligomers, enabling it to exist in an inactive conformation^39–41^. Hence, inhibitory oligomerization seems to be a conserved autoinhibition mechanism used by certain NLRs in different kingdoms of life. This mechanism ensures the prevention of accidental NLR activation at elevated concentrations and provides an explanation for constitutive high expression of some NLR genes without triggering autoactivation^5^. Constitutive accumulation of NLRs may prepare plants for a rapid response to pathogen infection. However, it remains to be investigated whether the mechanism of inhibitory oligomerization holds true for plant NLRs other than *Sl*NRC2. In particular, the residues responsible for mediating *Sl*NRC2 dimerization and oligomerization are not conserved in *Sl*NRC3 and *Sl*NRC4 (Extended Data Fig. 8b). Whether these two NRCs use inhibitory oligomerization or other mechanisms in addition to intradomain mediated autoinhibition to avoid autoactivation is unclear. Interaction with other proteins could be a potential mechanism, as observed in the inhibition of the prototype NLR MalT by MalY in *Escherichia coli*^42^. Our finding that *Sl*NRC2 oligomer-disrupting mutants enhance Rx-triggered disease resistance to PVX infection in systemic leaves suggests a potential trade-off between prevention of NLR autoactivation through inhibitory *Sl*NRC2 oligomerization and the efficacy of the *Sl*NRC2-mediated immune response. However, as disease resistance assays were performed here in *nrc2/3/4 N. benthamiana* triple mutant plants and *Sl*NRC2, *Sl*NRC3 and *Sl*NRC4 redundantly contribute to Rx-mediated resistance to PVX^27^, it seems likely that a potential trade-off is offset by functional redundancy between these NRC members.

The activation of *Sl*NRC2 may involve the relief of both inhibitory intradomain and intermolecular interactions through transient binding to Rx^36,38^, which might result in the formation of a primed state similar to the activation of ZAR1 (refs. ^11,43^). These events lead to the assembly of presumably hexameric *Sl*NRC2 resistosomes as demonstrated for *Sl*NRC4 (ref. ^19^). It remains unknown whether other components are required to relieve the inhibitory oligomerization of *Sl*NRC2. Further investigations are needed to fully comprehend how this new layer of inhibition is relieved and what subsequently triggers the activation of these NLRs. Nevertheless, our data suggest that plants have evolved several layers of inhibition to tightly regulate NLR activation and prevent unnecessary immune responses.

Unexpectedly, the cryo-EM structures of *Sl*NRC2 show that an IP molecule, primarily IP_6/5_, binds between the WHD and LRR domains. As the relative positions of these two structural domains remain unchanged during NLR activation^9^, this structural observation suggests that IP_6/5_ might constitutively bind *Sl*NRC2. *Sl*NRC2 protein purified from plants constitutively contained these two IP molecules, suggesting that no induction is required for the IP-binding activity of *Sl*NRC2. However, the possibility remains that IP_6_ and IP_5_ levels are altered in response to biotic or abiotic stimuli to modulate CP-induced and NRC2-dependent immune signalling. The biological relevance of the IP-binding activity is supported by the finding that mutations at the IP-binding site of *Sl*NRC2 compromised or abolished CP-induced cell death in *N benthamiana*. A role of IP_6_ in pattern-triggered immunity has been demonstrated previously^44^, although the mode of action and cellular targets of IP_6_ in plant defence remained undefined. The IP-binding site identified in this work is conserved among NRCs. Notably, the IP_6/5_-binding site is located on the inner surface of the C-terminal *Sl*NRC2 LRR domain, where effector proteins typically bind for activation of plant NLRs. Given the essential role of IP_6/5_ binding in CP-induced cell death, these data suggest that IP_6/5_ may function as a cofactor of *Sl*NRC2 in recognizing activated Rx. A similar role of IP_6_ has been demonstrated in auxin receptors, in which the IP acts as structural cofactor required for auxin to function as molecular glue enhancing TIR1–substrate interactions^45^. Like *Sl*NRC2, the auxin receptor TIR1 can also bind IP_5_ besides IP_6_ (ref. ^46^). We propose that IP_6/5_-binding to *Sl*NRC2 has a similar role in enhancing the interaction between *Sl*NRC2 and CP-activated Rx.

The finding that IP_6/5_ binding is crucial for CP-induced immune signalling of *Sl*NRC2 suggests a connection between IP signalling and plant defence responses, adding a new layer of complexity to the intricate network of interactions involving NLRs, effectors and other signalling components in plant immunity. IPs are known to participate in numerous cellular processes in plants and animals, including phosphate homeostasis, energy metabolism and signal transduction^47^. Understanding the interplay between IPs and NLRs will not only provide insights into the signalling mechanisms underlying plant defence responses but also shed light on the intricate interplay between immune signalling, cellular metabolism and homeostasis. In the latter scenario, it is important to investigate whether other metabolites or small molecules can play a cofactor role similar to IPs in NLR perception of pathogens.

## Methods

### Construct preparations

The insect cell codon-optimized DNA sequence of tomato *NRC2* (*SlNRC2* with the accession number: Solyc10g047320 in the Sol Genomics Network (SGN) database) was synthesized by Genewiz and recombinated into a modified *pFastBac1* vector with an N-terminal maltose-binding protein tag followed by a PreScission protease cleavage site. All mutants expressed in insect cells were cloned into the same vector as the wild-type *SlNRC2*.

### Protein expression and purification

The wild-type and mutants of *Sl*NRC2 were expressed in Sf21 cell line (Invitrogen). The cell line was not independently authenticated and was not tested for mycoplasma contamination. The proteins were purified by Dextrin resin (Smart-Lifesciences) and subjected to size exclusion chromatography (SEC). A total of 600 ml of Sf21 cells (Invitrogen) were infected with 15 ml of *NRC2* baculovirus and harvested 2 days after infection. Cell pellets were resuspended with 60 ml of lysis buffer (25 mM Tris-HCl pH 8.0, 150 mM NaCl, 1 mM dithiothreitol) and sonicated twice. After centrifugation, the supernatant was loaded onto a gravity column prepacked with 2 ml of Dextrin resin, then washed with 10 column volumes of lysis buffer and finally eluted with 10 ml of elution buffer (25 mM Tris-HCl pH 8.0, 150 mM NaCl, 1 mM dithiothreitol, 20 mM maltose). The filament can be observed in the Dextrin eluate when checked by negative-staining transmission electron microscopy (TEM). The eluate was further concentrated with a 30 kDa MWCO Amicon Ultra Centrifugal Filter (Millipore) to 1.5 ml and centrifuged, then the supernatant was analysed using SEC through a HiLoad 16/600 Superdex 200 pg or Superose 6 Increase 10/300 GL column (Cytiva) with Buffer E (10 mM Tris-HCl pH 8.0, 150 mM NaCl, 1 mM dithiothreitol). The filament co-eluted with aggregates in the void volume checked by negative-staining TEM and the peak of putative dimer eluted in the 59.5 ml for the HiLoad 16/600 Superdex 200 pg and 15.5 ml for the Superose 6 Increase 10/300 GL column. The putative dimer fractions were pooled up and concentrated to 4 mg ml^−1^ (filament appeared during concentration which can be observed visually and checked by negative-staining TEM) and centrifuged. A total of 400 µl of supernatant was digested by PreScission protease at 4 °C overnight and centrifuged at 16,000*g* at 4 °C for 30 min, then the semitransparent filament pellet was washed twice and resuspended in 400 µl of SEC Buffer E. The supernatant was subjected to Superdex 200 Increase 10/300 GL column and the dimer eluted around 11.3 ml corresponding to 158–440 kDa. Fractions from 11 to 13 ml were pooled up, concentrated and centrifuged and the supernatant was used for cryo-EM grid preparation.

### Cryo-EM specimen preparation

The filament pellet was resuspended by 400 µl of SEC buffer and 4 µl was loaded onto a glow-discharged Quantifoil Au200 grid and blotted for 3 s using a Vitrobot Mark IV (Thermo Fisher Scientific). The dimer specimen was concentrated to 0.7 mg ml^−1^ and centrifuged, then 4 µl sample was loaded onto a glow-discharged Quantifoil Au200 grid and blotted for 3 s using a Vitrobot Mark IV. The filament and soluble particles were well-distributed in the holes when checked by a Glacios 2 Cryo-TEM (Thermo Fisher Scientific). The screened grids containing filaments and dimers were sent to data collection using a 300 kV Titan Krios and Krios G4 Cryo-TEM (Thermo Fisher Scientific), respectively.

### Cryo-EM data collection

Micrographs of *Sl*NRC2 filaments were collected with Titan Krios microscope operated at 300 kV, equipped with Gatan K3 Summit direct electron detector equipped with a Cs corrector and a slit width of 20 eV on the Gatan Quantum energy filter. Stacks were automatically recorded using AutoEMation in super-resolution mode^48^. A nominal magnification of ×64,000 was used for imaging the samples, corresponding to a final pixel size of 1.0979 Å on image. Defocus values varied from −1.0 to −2.0 μm. The exposure time for both datasets was 2.56 s dose-fractionated into 32 subframes, leading to a total electron exposure of approximately 50 electrons per Å^2^ for each stack.

The NRC2 dimer and tetramer datasets were collected using a Titan Krios G4 (Thermo Fisher Scientific) equipped with a BioQuantum GIF energy filter with a slit width of 10 eV (Gatan) and a K3 direct detector (Gatan). All video stacks were automatically acquired at a magnification of ×105,000 under super-resolution mode with a calibrated physical pixel size of 0.85 Å. The total dose was 50 electrons per Å^2^ for each stack. Each video was fractionated into 40 frames with an exposure time of 2.89 s. Defocus values varied from −1.0 to −2.0 μm. Extended Data Table 1 summarizes the model statistics.

### Cryo-EM data processing of *Sl*NRC2 dimer and tetramer

A total of 4,149 micrographs were 2 × 2 binned, generating a pixel size of 0.85 Å per pixel. Motion correction and contrast transfer function (CTF) estimation were calculated by CryoSPARC^49^. A total of 2,258,817 particles were automatically picked using blob picking in CryoSPARC. After several rounds of 2D classification, 1,145,822 good particles were selected to generate ab initio models. The best initial model was used as the reference map for subsequent global 3D classification. After several rounds of 3D classification, 611,661 particles of NRC2 dimer were subjected to heterogeneous refinement with *C*_2_ symmetry and then 560,935 particles were selected for further homogeneous refinement. The final reconstruction map resulting in a resolution of 2.84 Å. Similarly, 159,294 particles of NRC2 tetramer were selected for further heterogeneous refinement with *C*_2_ symmetry. Finally, 154,084 good particles were subjected to homogeneous refinement using CryoSPARC. The final improved reconstruction map was refined to 3.17 Å. The resolution was determined according to the gold-standard Fourier shell correlation 0.143 criteria with a high-resolution noise substitution method. Local resolution distribution was evaluated using CryoSPARC. Detailed workflow was shown in Extended Data Fig. 2.

### Cryo-EM data processing of *Sl*NRC2 filament

The raw stacks of *Sl*NRC2 filament were motion-corrected by MotionCor2 and binned twofold^50^. CTF parameters of dose-weighted micrographs were determined using CTFFIND4 (ref. ^51^). Postmotion-corrected images were loaded into RELION (3.08 and later on 4.0 during last few rounds of 3D refinement)^52–54^. An auto-picking script was used to facilitate auto-picking filaments, with manual interventions to avoid cross junctions (https://github.com/Alexu0/Cryo-EM-filament-picker). Experimental helical segment extractions with various dimensions were tested on the remaining good helical sections. After testing 256 pixel, 400 pixel, 512 pixel boxes, 256 pixel box bin 2 and bin 1 settings showed the best helical layer lines. Eventually, particles of 256 pixel size were extracted from all the filaments, with about 70 Å step size. At 2D classification stage, helical settings were used to help align the particles and the best 1,074,191 particles were selected for further analysis, with good coverage of side views.

The parameters for the helical symmetry were determined through the analysis of the power spectrum of 2D class averages using RELION 3.08, following a similar approach used for determining the helical parameters of L7–TIR complexes^30^. The parameters were determined to be about 65 Å in the helical rise and about ±55° in the helical rotation. Specifically, the analysis showed that when segments were extracted using 256 pixel boxes, the reciprocal layer line analysis of the 2D averages indicated the presence of a 512/52 × 1.1 pixel repeat pattern along the helical axis. This indicated that the helix had a minimal helical rise of 10.83 Å or its integer *n* times of values. On the basis of the knowledge of the presence of a *C*_3_ symmetry, we tested three, six and nine times of 10.83 Å and determined that about 65 Å was the solution (*n* = 3 and 9 yielded irregular densities). There was also an obvious 140 Å helical pitch (2D pattern repeated every 140 Å along the helical axis), which indicated that for every 140 Å linear translation along the axis, the same molecular would rotate back to a same helical rotational angle. With an extra *C*_3_ symmetry, this indicated that for every 140 Å along the helical axis, every molecule rotated 120° or 120′ integer times. We then tested ±120/(140/65) = ±55° as the candidate helical rotation values. Because our calculation was based on 2D images, this helical rotation value could be both positive and negative values.

During the 3D classification runs, a cylindrical Gaussian noise density with diameter of 220 Å was used as initial model. After iterative 3D runs, with initially 15° rotational step size and 5 pixel step size with a 30 pixel range, then gradually narrowed down to 3.7° step size and 1 pixel step size, we obtained several 3D classes with good structural features. Helical parameters of 65 Å + −2 Å for the helical rise and −55° + −1.5° for the helical rotation were used to restrain helical search. Lastly, 280,426 particles with apparently more coherent helical parameters were used for final 3D refinement. The helical symmetry converged to 64.39 Å and −55.24°. So far, only *C*_1_ helical symmetry was applied.

In the following 3D refinement steps, a *C*_1_ symmetry density map was refined to around 4.25 Å, which also served as an unbiased reference of the final density. *C*_3_ symmetry was subsequently introduced in further 3D classifications, using the same 280,426 particles. This 3D class was then refined with tightened parameters, 1.8° rotational steps and 1 pixel linear step size. Then final high-resolution density map was obtained by iterative masking and CTF refinements and polishing (RELION 3.08 initially and repeated using RELION 4.0). The final map is verified to be 3.6 Å measured with gold-standard FSC curve (3.2 Å at the filament core, 4.5 Å at the 200–220 Å rim), using the Electron Microscopy Data Bank (EMDB) validation server. The detailed workflow is shown in Extended Data Fig. 2. The map has been deposited with a code of EMD-38685.

### Model building and refinement

The model of *Sl*NRC2 monomer predicted by AlphaFold2 was docked into the reconstruction map of *Sl*NRC2 dimer (Protein Data Bank (PDB) code 8XUO) and then manually adjusted in COOT^55–57^ followed by PHENIX^58^ refinement in real space with secondary structure and geometry restraints. The final refined *Sl*NRC2 dimer was docked into the cryo-EM density maps of the tetramer (PDB code 8XUQ) and filaments (PDB code 8XUV) followed by PHENIX refinement. ADP and IP_6_ were manually added using COOT. The final models after refinement were validated using EMRinger in the PHENIX package^59^. Statistics of the map reconstruction and model refinement can be found in Extended Data Table 1.

### Detection of IPs bound in insect cell-purified *Sl*NRC2

The standard used was d-myo-inositol 1,2,3,4,5,6-hexakisphosphate, dodecasodium salt (IP_6_) (Sigma) at a concentration of 100 µM. Subsequently, 50 µl of the insect cell-purified *Sl*NRC2 sample treated with SEC was mixed with 150 µl of methanol and left to incubate overnight. The supernatant was collected after centrifugation (13,000 rpm), followed by freeze-drying. Then, 30 µl of 1 mM HCl in 10% methanol solution was added for reconstitution, 5 µl of extracted solution was injected for analysis, whereas 1 µl of the standard was used for sample injection. LC–MS/MS analysis was conducted using Waters synapt XS QTof mass spectrometer connected with Waters UPLC system equipped with an Oligonucleotide BEH C18 Column (130 A, 1.7 µm, 2.1 ×100 mm^2^, Waters). The column temperature was maintained at 60 °C, whereas the sample tray was set at 4 °C. For the elution, phase A comprised 15 mM TEA and 400 mM HFIP, whereas phase B was a mixture of 50% phase A and 50% methanol. The flow rate was set at 0.2 ml min^−1^. Phase A was initially held at 95% for 1 min, followed by a linear decrease from 95% to 5% over 9 min. This was sustained at 5% for 2 min, followed by a rapid transition from 5% back to 95% within 0.1 min and then maintained at 95% for a further 3 min. The mass spectrometer was operated in negative mode and the MS parameters used were as follows: capillary voltage, 2 kV; sample cone, 30 V; source offset, 10 V; source temperature, 120 °C; desolvation temperature, 600 °C; nebulizer gas, 6 bar; cone gas flow rate, 50 l h^−1^; and desolvation gas flow rate 800 l h^−1^. A full scan spectrum was acquired with a mass range from *m*/*z* 50 to 1,000. For MS/MS spectra, the collision energy was set at 20 eV.

For native MS analysis, the *Sl*NRC2 protein concentration was adjusted to around 4 μM in 10 mM Tris-HCl pH 8.0, 100 mM NaCl, 1 mM dithiothreitol and 2 μl of the protein solution was injected for molecular weight determination, which was performed on a Thermo Vanquish UHPLC system coupled to a Thermo Q Exactive UHMR mass spectrometer. The LC separation was carried out on a Waters BEH SEC column (2.1 × 150 mm^2^, 1.7 μm) at room temperature, isocratic separation was implemented using 100 mM ammonium acetate pH 6.8 as the mobile phase with the flow rate at 0.2 ml min^−1^. The MS parameters used were as follows: capillary voltage, 3,500 V in positive mode; resolution, 12,500; in-source collision-induced dissociation voltage, 0 eV; IST voltage, −75 V; capillary temperature, 275 °C; sheath gas, 20; auxiliary gas, 5. Full scan spectra were acquired with a mass range from *m*/*z* 2,000 to 12,000 and the molecular weights of the proteins were deconvoluted and calculated by BioPharma Finder 4.0.

### Sequence alignments

Sequence motif logos to analyse the conservation of residues involved in IPx binding were created by means of WebLogo (https://weblogo.berkeley.edu/logo.cgi) using 134 previously published NRC sequences as an input^60^.

### Expression and purification of recombinant *Sl*NRC2 from transiently transformed leaves of *N. benthamiana*

The coding sequence of *SlNRC2* was inserted by means of the Gateway cloning system into a modified sequence of the expression vector *pGWB402*. The resulting sequence was driven by an enhanced 35S promoter followed by an N-terminally-tagged Twin-Strep epitope tag. DNA of the expression vector was transformed into the *A. tumefaciens* strain GV3101 pMP90 and grown on plates containing appropriate antibiotics for 48 h. Transformed colonies were grown in Luria–Bertani (LB) broth overnight, pelleted and resuspended in infiltration buffer (10 mM MES pH 5.6, 10 mM MgCl_2_ and 500 μM acetosyringone) to a final optical density OD_600_ of 1. Leaves of wild-type *N. benthamiana* plants were infiltrated and incubated in standard growing conditions for 48 h until harvest when the tissue was flash-frozen in liquid nitrogen.

A total of 200 g of frozen leaf tissue was ground in a mortar and pestle to a fine powder. All subsequent steps were performed at 4 °C. The pulverized tissue was slowly added to lysis buffer (50 mM Tris-HCl pH 8.0, 150 mM NaCl, 10 mM dithiothreitol, 5% glycerol, 0.5% Tween-20, 5% BioLock (IBA Lifesciences) and four vials of Protease Inhibitor Mix P (Serva Electrophoresis) and filled to 400 ml with ultrapure water). The lysate was clarified twice by centrifugation at 30,000*g* and passed through two layers of Miracloth (Merck Millipore). A total of 1 ml of Strep-Tactin XT Sepharose chromatography resin (Cytiva) was washed in 14 ml of wash buffer (50 mM Tris-HCl pH 8.0, 150 mM NaCl, 2 mM dithiothreitol and 0.1% Tween-20 and filled to 400 ml with ultrapure water) and collected by centrifugation at 100*g* for 2 min. The resin was added to the lysate and rotated end-over-end for 2 h. The resin was collected by centrifugation at 100*g* for 3 min and washed three times with wash buffer. A total of 1 ml of elution buffer (wash buffer + 50 mM biotin) was added to the resin and rotated end-over-end for 30 min. Elution was repeated a total of five times. The 5 ml of eluate was concentrated with a 30 kDa cut-off centrifugation concentrator to 500 μl. The concentrated eluate was further analysed through SEC by loading it on a Superose 6 Increase 10/300 GL column and run at 0.3 ml min^−1^. The elution fractions were analysed by loading 45 μl/500 μl onto SDS–PAGE and stained with Coomassie brilliant blue.

### Sample preparation and LC–MS detection of IPs (IPx) in plant-purified *Sl*NRC2

The method was adopted from ref. ^61^ with modifications^61^. Authentic standards of d-*myo*-inositol 1,2,3,4,5,6-hexakisphosphate, dodecasodium salt (IP_6_) (Sigma), d-*myo*-inositol 1,2,3,4,5-pentakis-phosphate decasodium salt (IP_5_) (Sigma), d-*myo*-inositol 1,4,5-tris-phosphate trisodium salt (IP_3_) (Sigma) and d-*myo*-inositol 1-monophosphate dipotassium salt (IP_1_) (Sigma) were used as reference chemicals. Either 50 µl of sample or 10 mM standard chemical were lyophilized (RVC 2-18 CDplus concentrator, Martin Christ) and dissolved in 100 µl of 1 mM HCl in methanol. For methylation, 100 µl of 2 M (trimethylsilyl) diazomethane (TMSD, Thermo Scientific Chemicals) was added. The methylation reaction was stopped after 10 min by adding 5 µl of glacial acetic acid (Merck). Using pressured air, the stopped reaction was dried (XcelVap, Analytica One Company). Dried methylated sample and standard was dissolved in 100 µl of 10% methanol and ready for LC–MS. Samples were normalized using protein concentration to adjust chromatography injection volume.

Chromatography was performed on a Nexera XR 40 series HPLC (Shimadzu) using a Nucleodur Sphinx RP, 3 µm, 150 × 2 mm (Macherey Nagel). The column temperature was maintained at 40 °C and the sample tray at 4 °C. Samples (variable 4–33 μl) were injected at a flow rate of 0.4 ml min^−1^ using 10 mM ammonium formate pH 4.2 and methanol as mobile phases A and B, respectively. Metabolites were eluted using the 12 min gradient profile 0 min, 2% B; 0–6 min, 100% B; 6–8 min, 100% B; 8–8.1 min, 2% B. The LC–MS-8060 triple quadrupole mass spectrometer with electrospray ionization (Shimadzu) was operated in positive mode. Scheduled MRM was used to monitor analyte parent ion to product ion formation. MRM conditions were optimized using methylated authentic standard chemicals (MeIPx) including: MeIP_6_ ([M + H] 828.90 > 451.15, 828.90 > 357.05, 828.90 > 325.15), MeIP_5_ ([M + H] 720.90 > 311.10, 720.90 > 563.05, 720.90 > 217.15), MeIP_3_ ([M + H] 505.10 > 127.15, 505.10 > 473.10, 505.10 > 109.15), MeIP_1_ ([M + H] 289.10 > 127.15, 289.10 > 109.20, 289.10 > 95.15). Both Q1 and Q3 quadrupoles were maintained in unit resolution. LabSolutions LC–MS v.5.118 software was used for data acquisition and LabSolutions Postrun for processing (both Shimadzu). Metabolites were quantified by scheduled MRM peak integration. TMSD, 2 M solution in hexanes; methanol, water and ammonium formate had LC–MS quality.

### Generating *NRCs* knockout mutants using CRISPR–Cas9 in *N. benthamiana*

*The nrc2/3/4* knockout mutant was generated using the CRISPR–Cas9 system as previously described^62^.The guide RNAs that target *NRC2*, *NRC3* and *NRC4* were designed near the N terminus of *NRC* genes (oligonucleotides are shown in Supplementary Table 1) and were cloned to shuttle vectors *pDGE332*, *pDGE333*, *pDGE335*, *pDGE336*, *pDGE495* and *pDGE497* through BpiI cut/ligation reaction, respectively. Derivatives of shuttle vectors, loaded with single guide RNA sequences, were used for assembly in recipient vector *pDGE311* (*nptII-Bs3-Cherry-35S:Cas9-ccdB*) by BsaI cut/ligation to generate a final construct for *N. benthamiana* transformation. The recipient vector *pDGE311* containing six sgRNAs targeting the *NRC2*, *NRC3* and *NRC4* genes was transformed to *A. tumefaciens* GV3101 pMP90RK through electroporation.

*N. benthamiana* plants were transformed as previous described^63^ following an online protocol (10.17504/protocols.io.sbaeaie). T_0_ transgenic plants were selected in the medium with kanamycin (100 mg l^−1^) and then transferred into the soil. Genome DNA was extracted from transgenic plants by the CTAB method and genotyped using PCR amplification with the respective primers (Supplementary Table 1). The amplified DNA fragments were sequenced and compared to the sequence of wild-type *N. benthamiana*. In T_2_ transgenic lines, it would be more efficient to isolate the *NRC*s knockout mutants by selecting the plants that without hypersensitive response after infiltration of *Rx* and *CP* elicitor. The homozygous *nrc2/3/4* (simultaneous knockout of *NRC2*, *NRC3* and *NRC4*), non-transgenic seed lots were used for experiments.

### Site-directed mutagenesis of *SlNRC2* for in planta analyses

The complementary DNA of insect cell codon-optimized *SlNRC2* without stop codons was amplified by PCR using attB primers followed by BP reaction (Gateway) and cloned into *pDONR207* by recombination (Invitrogen) to generate a Gateway-compatible entry clone. The *pDONR207-SlNRC2* was used as template to generate the indicated mutations through PCR mutagenesis using the Q5 Site-Directed Mutagenesis Kit (NEB). Sequences of oligonucleotides are provided in Supplementary Table 1. LR-Clonase II (Thermo Fisher) was used to recombine the genes into the modified expression vector *pGWB402* (aforementioned C-terminal Twin-Strep-HA tag), *pXCSG-HA-StrepII* (C-terminal HA-StrepII tag), *pGWB411* (C-terminal FLAG tag) and *pK7FWG2.0* (C-terminal eGFP tag). All constructs were verified by DNA sequencing. Generated expression constructs were transformed into *A. tumefaciens* GV3101 pMP90RK through electroporation.

### Cell death assays in *N. benthamiana*

Wild-type and *nrc2/3/4 N. benthamiana* plants were cultivated in a greenhouse at 23 °C, under a long-day (16 h light/8 h dark) photoperiod. For hypersensitive-response assay, 4–5-week-old *nrc2/3/4 N. benthamiana* plants were used. The *A. tumefaciens* strain GV3101 pMP90RK containing the indicated constructs together with *P19* suppressor were infiltrated into leaves. Final OD_600_ of all *A. tumefaciens* suspensions were adjusted in infiltration buffer (10 mM MES, 10 mM MgCl_2_ and 150 μM acetosyringone, pH 5.6). For the cell death assay, we used final OD_600_ = 0.2 for *Rx-HA-StrepII*, *SlNRC2-eGFP variants*, OD_600_ = 0.1 or 0.05 of *CP-FLAG* for IP_6/5_ binding mutants and oligomer-disrupting mutations, respectively. After agro-infiltration, *N. benthamiana* plants were placed in a controlled growth chamber, under a 16 h light/8 h dark regimen at 23 °C. Hypersensitive-response phenotype photos were observed and photographed 3 or 5 days after infiltration. Images of agrobacteria-infiltrated leaf spots were taken at 3 days (for IP_6/5_ binding mutants) to 5 days (for oligomer-disrupting mutants).

The ion leakage assay was performed as described previously^64^ with slight modifications. After agro-infiltration, *N. benthamiana* plants were placed under a 16 h light/8 h dark growth chamber at 23 °C. The 6 mm leaf discs from *N. benthamiana* agroinfiltrated leaves were taken at 32 h (for IP_6/5_ binding mutants) or 40 h (for oligomer-disrupting mutations) after infiltration. The leaf discs were washed in 15 ml of Milli-Q water (18.2 MΩ × cm) for 30–60 min, transferred to a 48-well plate with 0.5 ml of Milli-Q water in each well and incubated in a growth chamber with light on. Ion leakage was measured at 8 h with a Horiba Twin Model B-173 conductometer. For statistical analysis, results of measurements for individual leaf discs (five biological independent samples were tested for one experiment) were combined from three independent experiments. One-way ANOVA was used and significantly different values were labelled with different letters (adjusted *P* < 0.05).

### Co-immunoprecipitation assay in *N. benthamiana*

Co-immunoprecipitation assays were performed as described previously^65^, with slight modifications. Briefly, *A. tumefaciens* strain GV3101 pMP90RK containing the indicated constructs was co-infiltrated into 4–5-week-old *N. benthamiana* leaves. The samples were harvested at 48 h after infiltration. Samples were ground into powder using liquid nitrogen and homogenized in protein extraction buffer (10% glycerol, 50 mM Tris-HCl pH 7.5, 150 mM NaCl, 0.5 mM EDTA, 5 mM dithiothreitol, 0.2% NP-40, 1 mM NaF and 20 µM MG132 with 1× Roche protease inhibitor cocktail). The extract was centrifuged twice at 4 °C, 12,000*g* for 15 min. After protein extraction, 20 µl of GFP-trap agarose beads (ChromoTek, GTMA) were added to the samples and incubated with rotation at 4 °C for 3 h. The precipitated samples were centrifuged at 1,000*g* for 3 min at 4 °C. The beads were washed three times with wash buffer (50 mM Tris-HCl pH 7.5, 150 mM NaCl, 0.5 mM EDTA, 5 mM dithiothreitol, 0.2% NP-40, 1× Roche protease inhibitor cocktail). Proteins were eluted by boiling the beads in 1× Laemmli sample buffer (Bio-Rad, 1610747) at 95 °C for 5 min. The protein samples were then separated by SDS–PAGE and analysed by immunoblotting. Antibodies used in this work were anti-GFP (Takara, 632569), anti-FLAG (Sigma, F1804) and anti-HA (Roche, C29F4).

### Transient gene expression in *N. benthamiana* and protein detection by immunoblotting

*Agrobacterium* harbouring the indicated constructs was infiltrated into the fully expanded leaves of 4–5-week-old *N. benthamiana* plants. To detect protein accumulation, leaf discs from three individual plants were collected 24 h after infiltration, flash-frozen in liquid nitrogen and ground into powder using a Tissue lyser (Qiagen). A 100 mg aliquot of plant tissue powder was resuspended in 200 μl of lysis buffer (10% glycerol, 50 mM Tris-HCl pH 7.5, 150 mM NaCl, 0.5 mM EDTA, 10 mM dithiothreitol, 0.2% NP-40, 0.1% Triton X-100, 1× Roche protease inhibitor cocktail) and was vortexed and then placed on ice for 20 min. After centrifugation twice at 12,000*g* for 15 min at 4 °C, 4× Laemmli sample buffer (Bio-Rad, 1610747) was added to the supernatant samples and the mixture was denatured at 95 °C for 5 min. Samples were loaded onto a 7.5% SDS–PAGE gel. Separated proteins were transferred to a PVDF membrane and probed with anti-GFP (Takara, 632569), anti-FLAG (Sigma, F1804) and anti-HA (Roche, C29F4).

### PVX infection assay for testing the *Sl*NRC2 variants in Rx-mediated resistance

The PVX inoculation was performed by infiltration of the *A. tumefaciens* strain GV3101 pSoup harbouring binary PVX-based expression vector^66^ (pSfinx) at a concentration of OD_600_ = 0.005 in the lower mature leaves (local leaves) of 5-week-old *nrc2/3/4* mutant. At the same time, *A. tumefaciens* strain carrying *Rx-FLAG* (OD_600_ = 0.3) and variants *SlNRC2-Twin-Strep-HA* (OD_600_ = 0.3) or empty vector were co-infiltrated in the upper leaves (systemic leaves). PVX spread from the local leaves to the *Rx-* and *SlNRC2-*expressing systemic leaves. At 7 days after infiltration, five 10 mm leaf discs from each condition were collected using a biopsy punch from the infiltrated areas in the systemic leaves infiltrated with *Rx* and *SlNRC2* variants. RNA was extracted from these samples using TRIzol RNA Isolation Reagents (Invitrogen). The relative expression of RNA encoding *CP* of PVX virus was normalized to the *N. bethamiana F-Box* gene, which was previously described^67^. The gene-specific primers are listed in the Supplementary Table 1.

### Blue Native–PAGE assays

To determine *Sl*NRC2 oligomeric status in vivo, we performed Blue Native–PAGE using the Bis-Tris Native–PAGE system (Invitrogen) according to the manufacturer’s instructions with minor modifications. Briefly, 30 µl of Strep-Tactin XT Sepharose chromatography resin (Cytiva) was added to the protein extract after centrifugation as described above. After 1 h incubation at 4 °C, the resin was collected by centrifugation at 1,000*g* for 3 min and washed three times with wash buffer (10% glycerol, 50 mM Tris-HCl pH 7.5, 50 mM NaCl, 2 mM dithiothreitol, 0.2% NP-40, 0.05% Triton X-100 and 1× Roche protease inhibitor cocktail). Then, 100 µl of elution buffer (wash buffer + 50 mM biotin) was added to the resin and followed by end-over-end rotation for 30 min. Sample aliquots for Blue Native–PAGE were spiked with Native–PAGE G-250 additive to a final concentration of 0.05% and placed on ice for 30 min before being loaded into the gel for electrophoresis. Protein samples and unstained Native Mark (Invitrogen, LC0725) were loaded and run on a Native–PAGE 3–12% Bis-Tris gel according to the manufacturer’s instructions. The proteins were then transferred to polyvinylidene difluoride membranes followed by immunoblot analysis with the desired antibodies.

### Reporting summary

Further information on research design is available in the Nature Portfolio Reporting Summary linked to this article.

## Online content

Any methods, additional references, Nature Portfolio reporting summaries, source data, extended data, supplementary information, acknowledgements, peer review information; details of author contributions and competing interests; and statements of data and code availability are available at 10.1038/s41586-024-07668-7.

## Supplementary information

Supplementary Information This file contains Supplementary Fig. 1 and Table 1.

Reporting Summary

## Source data

Source Data Fig. 3

Source Data Fig. 5

Source Data Extended Data Fig. 5

## Figures and Tables

**Extended Data Table 1:**
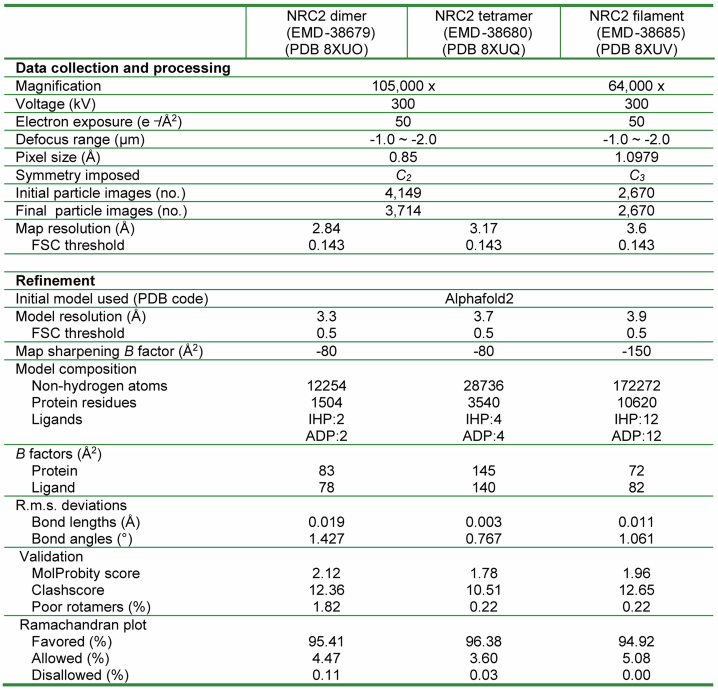
Cryo-EM data collection, refinement and validation statistics

The cryo-EM data of *Sl*NRC2 dimer and tetramer, and filament were collected and processed separately. Shown are the detailed statistics for the cryo-EM data collection, processing and refinement for *Sl*NRC2 dimer, tetramer and filament.

**Extended Data Table 2:**
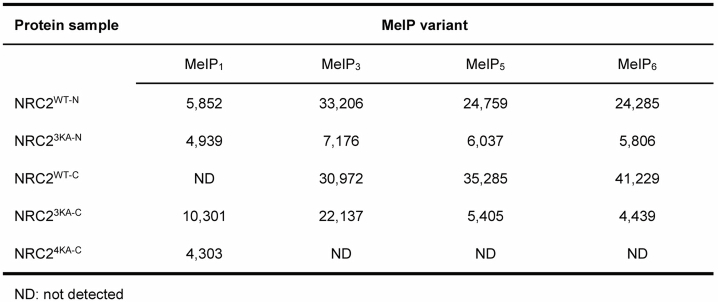
MeIPx detection in purified *Sl*NRC2 protein

Wild-type and triple/quadruple mutant versions of NRC2, either N- or C-terminally tagged, were expressed in *N. benthamiana* and subsequently purified. Equal amounts of protein were subjected to analysis. The numerical values represent the integration area of the MRM (Multiple Reaction Monitoring).

## Data Availability

All data are available in this article and its Supplementary Information. The atomic coordinates for the *Sl*NRC2 dimer, tetramer and filament have been deposited in the PDB with accession codes 8XUO, 8XUQ and 8XUV, respectively. The corresponding electron microscopy maps have been deposited in the EMDB with accession codes EMD-38679 (dimer), EMD-38680 (tetramer) and EMD-38685 (filament). Structures of inactive ZAR1 (PDB code 6J5W), ZAR1 resistosome (PDB code 6J5T), inactive *Sl*NRC1 NBD–HD1–WHD (PDB code 6S2P) and the *Sl*NRC1–SS15 complex (PDB code 8BV0) for alignment are obtained from PDB. The sequence of *Sl*NRC2 is available in the SGN database under accession number Solyc10g047320 and sequences of Rx and CP are available at GenBank under accession codes CAB50786 and CAA84016. Full version of gels and blots are provided in Supplementary Fig. 1. Primers used in this study are provided in Supplementary Table 1. Source data are provided with this paper.

## Code availability

No custom code was used in this study.
