# Supplementary material for: Oligomerization-mediated autoinhibition and cofactor binding of a plant NLR
Source: Nature. 2024 Jun 12;632(8026):869–76. doi: 10.1038/s41586-024-07668-7 (PMC11338831; doi:10.1038/s41586-024-07668-7)
Supplement: Supplementary file 1 — This file contains Supplementary Fig. 1 and Table 1. [file 41586_2024_7668_MOESM1_ESM.pdf]

nature portfolio

https://doi.org/10.1038/s41586-024-07668-7

Supplementary information

Oligomerization-mediated autoinhibition and cofactor binding of a plant NLR

In the format provided by the authors and unedited

Supplementary Figure 1: uncropped blots, gels images and pictures of tobacco leaves for HR phenotypes

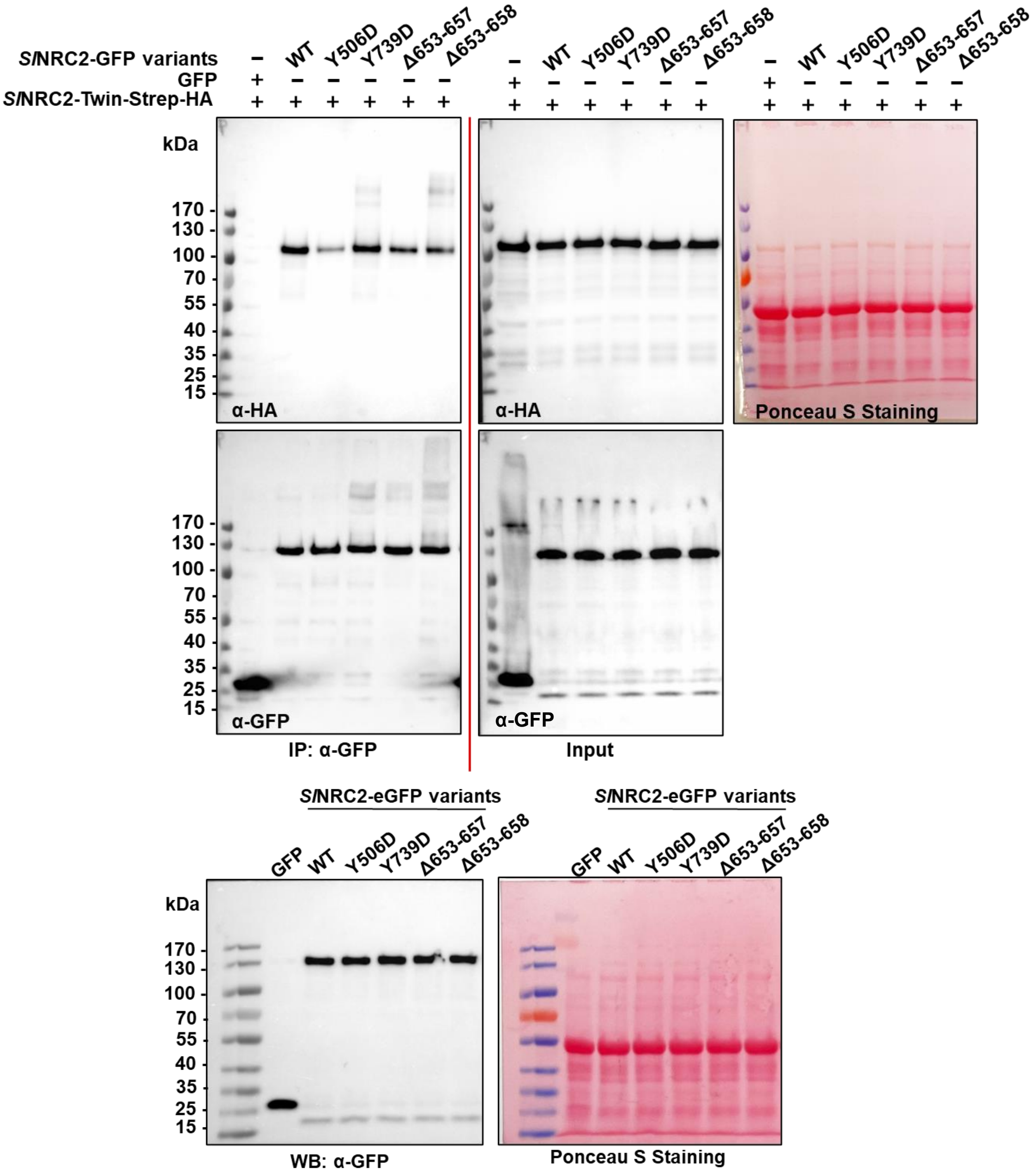

Uncropped western blot pictures corresponding to **Fig. 3c, d**.

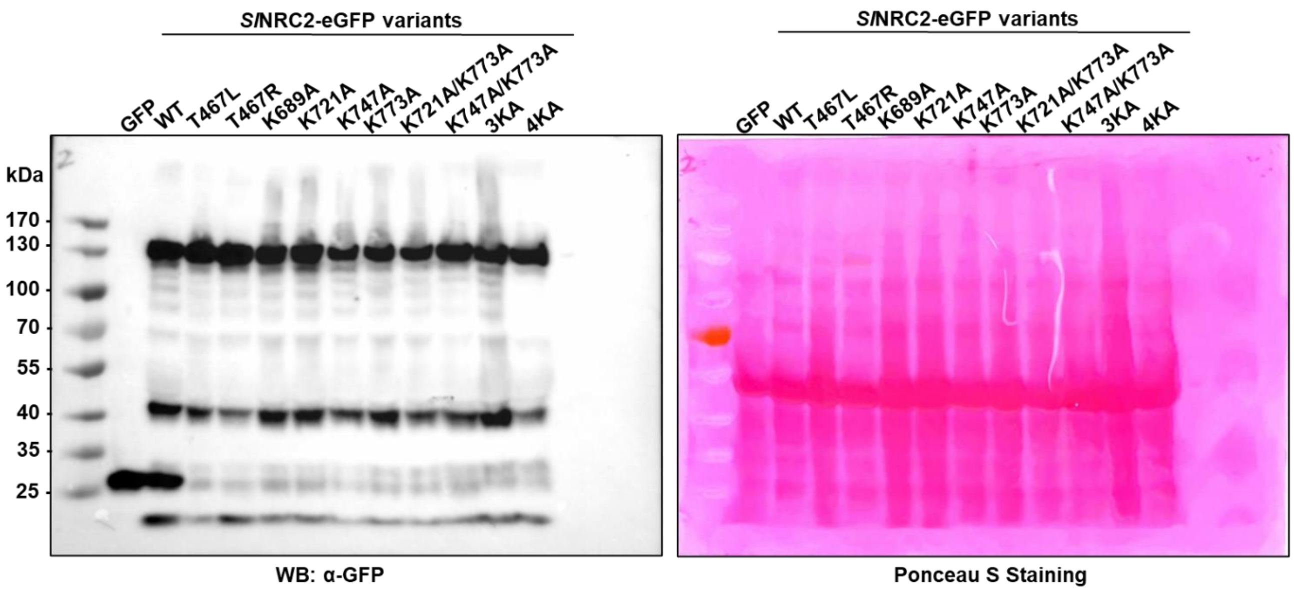

Uncropped western blot corresponding to **Fig. 5c**.

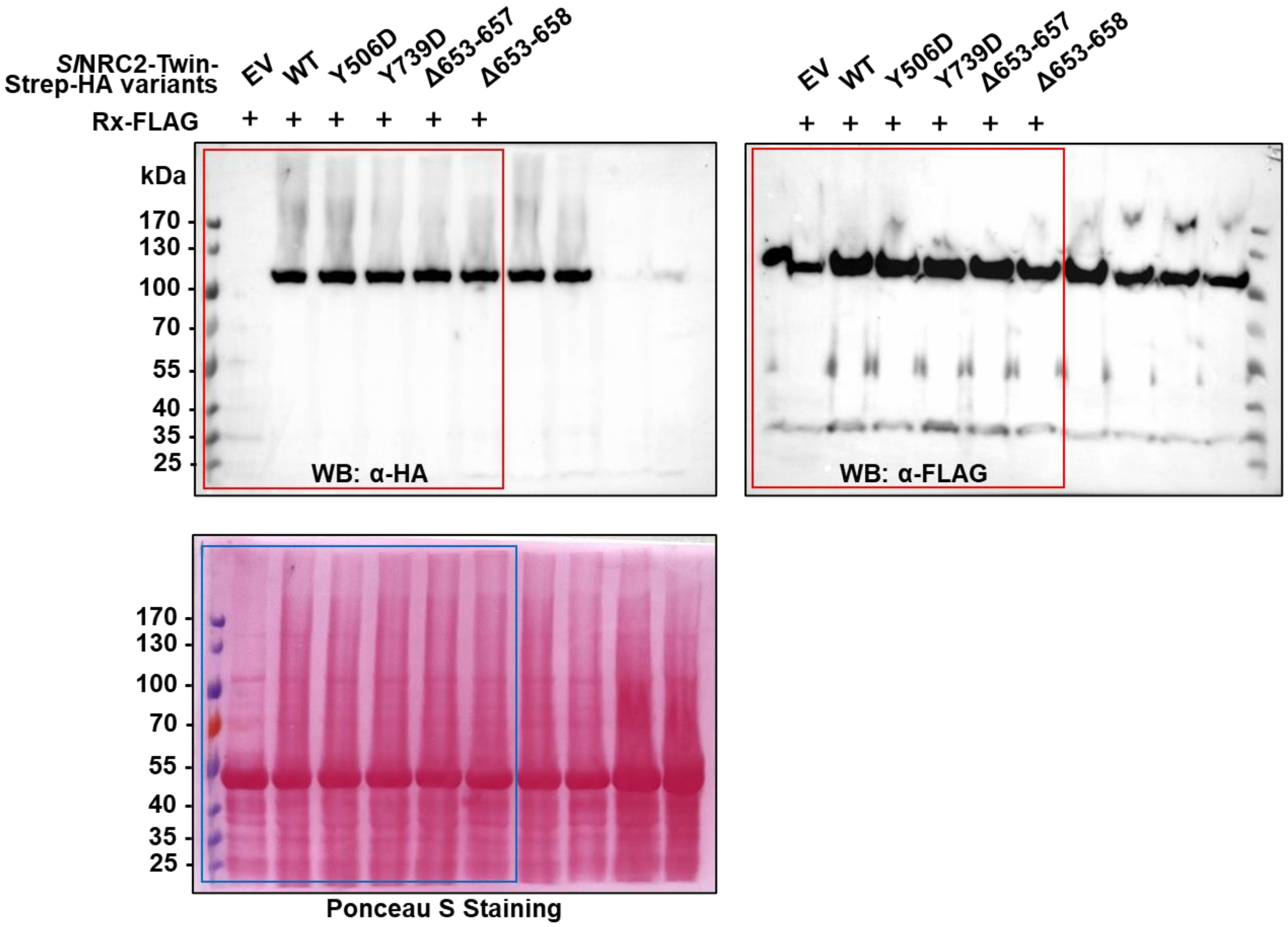

Uncropped western blot for the PVX assay corresponding to **Extended Data Fig. 5**. The areas marked by red and blue rectangles were used for this work.

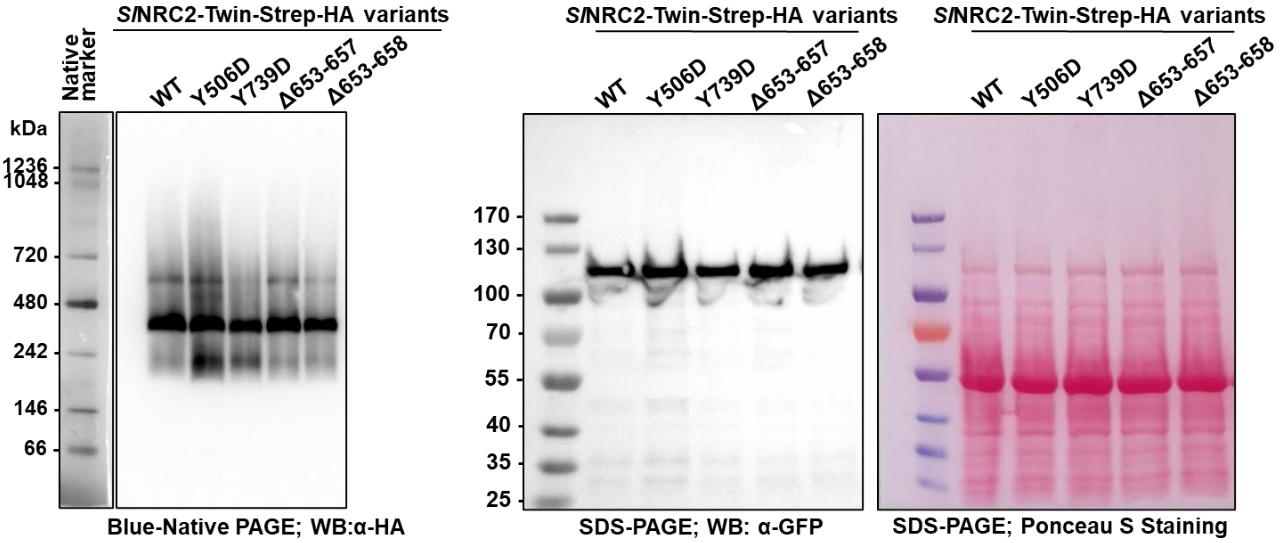

Supplemental Information 6. Uncropped western blot pictures for Blue-Native PAGE assay corresponding to **Extended Data Fig. 5.** For Ponceau S staining membrane, the same samples were run in a parallel gel.

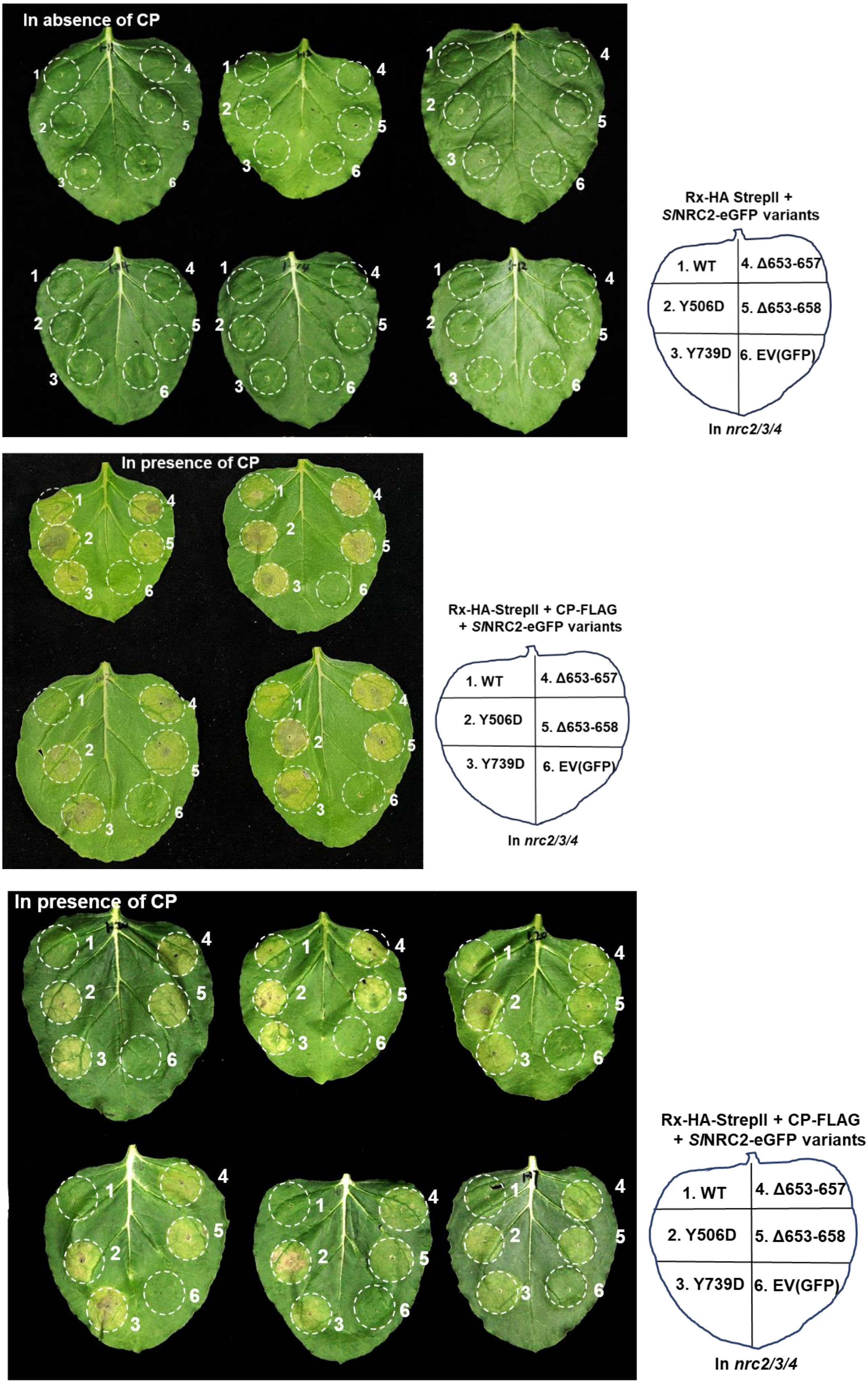

Rx-triggered cell death phenotypes that mediated by *Sl*NRC2 and *Sl*NRC2 oligomer-disrupting mutations in *Nicotiana benthiamiana.* Corresponding to **Fig. 3d**

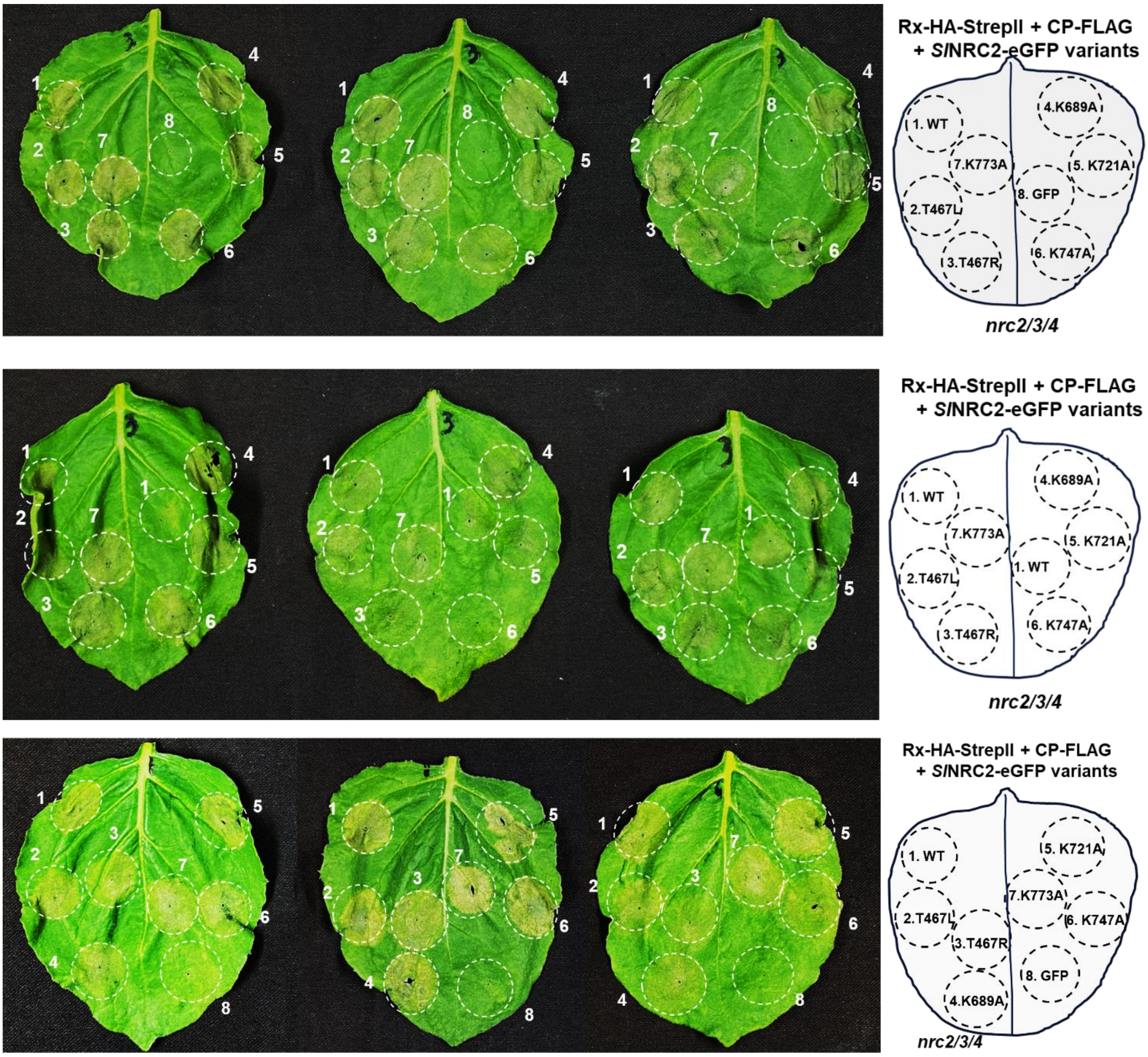

Rx-triggered hypersensitive response phenotypes of *Sl*NRC2 and *Sl*NRC2 IP binding mutants in *Nicotiana benthiamiana.* Corresponding to **Fig. 5c**. (To be continued)

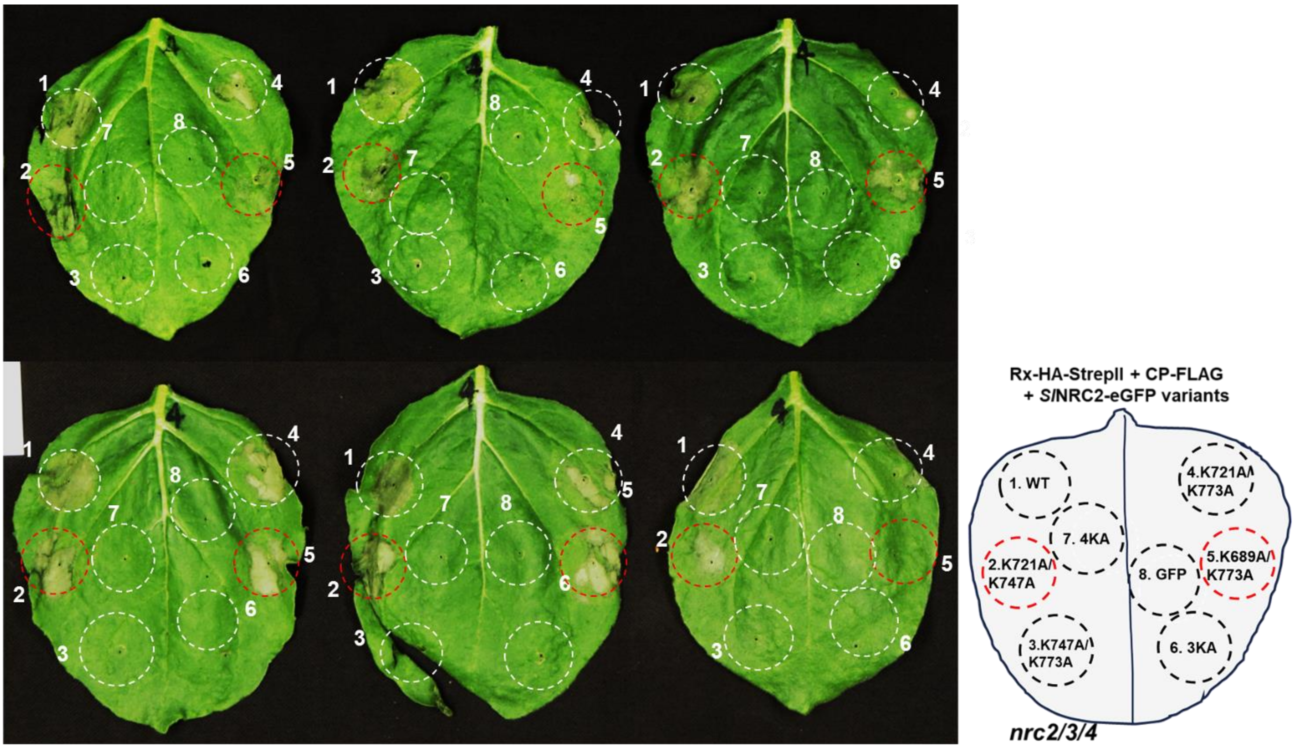

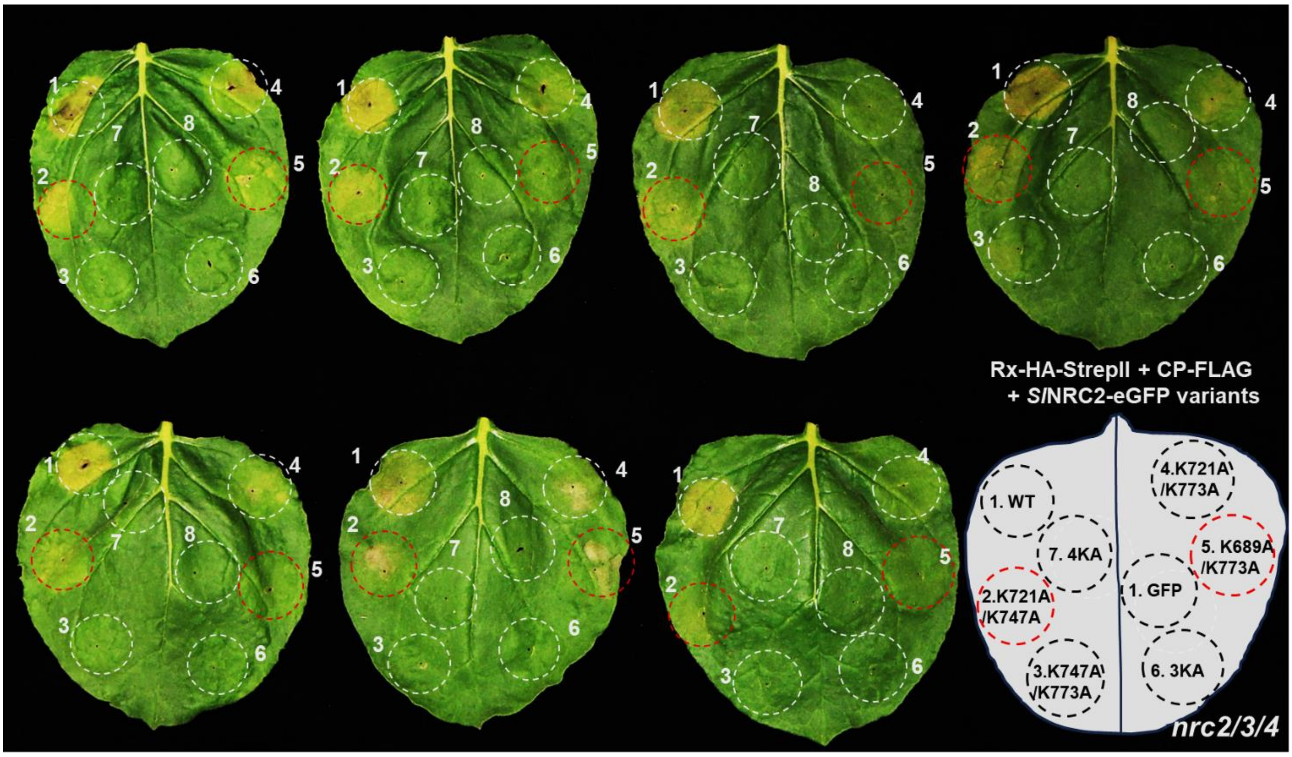

Rx-triggered hypersensitive response phenotypes of *Sl*NRC2 and *Sl*NRC2 IP binding mutants in *Nicotiana benthiamiana.* Corresponding to **Fig. 5c**. The cell death phenotypes of red circle labeled mutants are not presented in this work.

Supplementary Table S1 | Nucleotides used for constructions, mutagenesis, CRISPR editing and RT-qPCR.

**Primer name**	**Sequence**
SlNRC2-attB-F	GGGGACAAGTTTGTACAAAAAAGCAGGCTTCATGGCTAACGTGGCCGTGGAG
SlNRC2-attB-R	GGGGACCACTTTGTACAAGAAAGCTGGGTCGAGGTCGGGGGGGAAAATGG
Rx-attB-F	GGGGACAAGTTTGTACAAAAAAGCAGGCTTCATGGCTTATGCTGCTGTTAC
Rx-attB-R	GGGGACCACTTTGTACAAGAAAGCTGGGTCCTCGACATTATTGCGGCAAG
CP-attB-F	GGGGACAAGTTTGTACAAAAAAGCAGGCTTCATGACTACACCAGCCAACAC
CP-attB-R	GGGGACCACTTTGTACAAGAAAGCTGGGTCTGGGGGTAGTGAAACAACTG
*SlNRC2*_Y506D_R	CTGCTCGCTACCGAGTTTGATC
*SlNRC2*_Y506D_F	gACTTCCCCGGTAAACGTGAG
*SlNRC2*_Y739D_R	AGCGGGGGGGAGGCGCAGCT
*SlNRC2*_Y739D_F	gACATCTTCCCTACTAAGCTC
*SlNRC2*_1956_R	AGCCACGGGGACGGGCAGCT
*SlNRC2*_Δ653-657_F	GTGACTCTGGTGAACCAATC
*SlNRC2*_Δ653-658_F	ACTCTGGTGAACCAATCTCTG
*SlNRC2*_T467L_R	ACGTTCCATGACCATCACCAG
*SlNRC2*_T467R_R	AggTCCGACGGCCAGATCAAAAC
*SlNRC2*_T467L/R_F	ctCTCCGACGGCCAGATCAAAAC
*SlNRC2*_K689A_R	CTTGAGGTTGGGGGTGCGGG
*SlNRC2*_K689A_F	gcGCTGGGCATCCGTGGC
*SlNRC2*_K721A_R	CAGATTCTCCAGATACTCCAGAC
*SlNRC2*_K721A_F	gcGCTCATCAACGACAGCTC
*SlNRC2*_K747A_R	GCGGAGCTTAGTAGGGAAGATG
*SlNRC2*_K747A_F	gcACTCACTCTGCTGGATACTTG
*SlNRC2*_K773A_R	CAGCACCTCCAGATGTTCCAG
*SlNRC2*_K773A_F	gcGATGAAGGAGAACGGTTTCAG
sg*NRC2*_1F	attgAACGATGTCCACAAAGAAT
sg*NRC2*_1R	aaacATTCTTTGTGGACATCGTT
sg*NRC2*_2F	attgCTTCACAAGGACAAAGGGGT
sg*NRC2*_2R	aaacACCCCTTTGTCCTTGTGAAG
sg*NRC3*_1F	attgTCAAACAAGCAGCTAAATCA
sg*NRC3*_1R	aaacTGATTTAGCTGCTTGTTTGA
sg*NRC3*_2F	attgAATCACTAGTAAAGAAGATA
sg*NRC3*_2R	aaacTATCTTCTTTACTAGTGATT
sg*NRC4*_1F	attgAAAAACGGTACATACCGCAG
sg*NRC4*_1R	aaacCTGCGGTATGTACCGTTTTT
sg*NRC4*_2F	attgAGTCAGGAATCTTGCAGCTG
sg*NRC4*_2R	aaacCAGCTGCAAGATTCCTGACT
NbNRC2a-F	AGTCAACACCATCCATTTGCAC
NbNRC2a-R	TTGAAGGGCCTGAAGGTCAAG
NbNRC2b-F	AACACCACTCATTTGCGCTT
NbNRC2b-R	TCGACACCTTTGGCAGATTGA
NbNRC3-F	TATTGGTTGGCACTTGGCAG
NbNRC3-R	TCAGCAACCCCTTTTGCTCT
NbNRC4a-F	CCCACCTTTTATTCTTGAGGAA
NbNRC4b-F	CCACCTTATGTTCTTAGAGAG
NbNRC4-R	AACAGACCGAACCGAACCAT
PVX_CP-F	TTCGACTTCTTCAATGGAGTC
PVX_CP-R	TCCAGTGATACGACCTCG
Nb_F-box-F	GGCACTCACAAACGTCTATTTC
Nb_F-box-R	ACCTGGGAGGCATCCTGCTTAT
