# Supplementary material for: Oligomerization-mediated autoinhibition and cofactor binding of a plant NLR
Source: Nature. 2024 Jun 12;632(8026):869–76. doi: 10.1038/s41586-024-07668-7 (PMC11338831; doi:10.1038/s41586-024-07668-7)
Supplement: Supplementary file 2 — Reporting Summary [file 41586_2024_7668_MOESM2_ESM.pdf]

nature portfolio

Corresponding author(s): Jijie Chai
Paul Schulze-Lefert
Shoucai Ma

Last updated by author(s): May 22, 2024

Reporting Summary

Nature Portfolio wishes to improve the reproducibility of the work that we publish. This form provides structure for consistency and transparency in reporting. For further information on Nature Portfolio policies, see our Editorial Policies and the Editorial Policy Checklist.

Statistics

For all statistical analyses, confirm that the following items are present in the figure legend, table legend, main text, or Methods section.

n/a	Confirmed	
☐	☒	The exact sample size (*n*) for each experimental group/condition, given as a discrete number and unit of measurement
☐	☒	A statement on whether measurements were taken from distinct samples or whether the same sample was measured repeatedly
☐	☒	The statistical test(s) used AND whether they are one- or two-sided<br>*Only common tests should be described solely by name; describe more complex techniques in the Methods section.*
☒	☐	A description of all covariates tested
☐	☒	A description of any assumptions or corrections, such as tests of normality and adjustment for multiple comparisons
☐	☒	A full description of the statistical parameters including central tendency (e.g. means) or other basic estimates (e.g. regression coefficient) AND variation (e.g. standard deviation) or associated estimates of uncertainty (e.g. confidence intervals)
☐	☒	For null hypothesis testing, the test statistic (e.g. *F*, *t*, *r*) with confidence intervals, effect sizes, degrees of freedom and *P* value noted<br>*Give P values as exact values whenever suitable.*
☒	☐	For Bayesian analysis, information on the choice of priors and Markov chain Monte Carlo settings
☒	☐	For hierarchical and complex designs, identification of the appropriate level for tests and full reporting of outcomes
☒	☐	Estimates of effect sizes (e.g. Cohen's *d*, Pearson's *r*), indicating how they were calculated

Our web collection on statistics for biologists contains articles on many of the points above.

Software and code

Policy information about availability of computer code

Data collection	Titan Krios and Titan Krios G4 (Thermo Fisher Scientific), K3 Summit camera (Gatan)
Data analysis	EPU 2 (Thermo Fisher Scientific) 2.8.1.10REL<br>Relion 3.08<br>Relion 4.0<br>CryoSPARC<br>Coot 0.9<br>PHENIX 1.18.2<br>ChimeraX 1.15<br>Pymol Molecular Graphics System 1.7.2.1.<br>GraphPad Prism 8

For manuscripts utilizing custom algorithms or software that are central to the research but not yet described in published literature, software must be made available to editors and reviewers. We strongly encourage code deposition in a community repository (e.g. GitHub). See the Nature Portfolio guidelines for submitting code & software for further information.

Data

Policy information about availability of data

All manuscripts must include a data availability statement. This statement should provide the following information, where applicable:

- Accession codes, unique identifiers, or web links for publicly available datasets
- A description of any restrictions on data availability
- For clinical datasets or third party data, please ensure that the statement adheres to our policy

All data are available within this article and its Supplementary Information. The atomic coordinates for the SlNRC2 dimer, tetramer and filament have been deposited in the Protein Data Bank (PDB) with accession codes 8XUO, 8XUQ and 8XUV, respectively. The corresponding EM maps have been deposited in the Electron Microscopy Data Bank (EMDB) with accession codes EMD-38679 (dimer), EMD-38680 (tetramer) and EMD-38685 (filament), respectively. Validation reports for the SlNRC2 dimer, tetramer and filament are provided in Supplementary Table S2, S3, and S4. Structures of inactive ZAR1 (PDB code: 6J5W, https://www.rcsb.org/structure/6J5W), ZAR1 resistosome (PDB code: 6J5T, https://www.rcsb.org/structure/6J5T), inactive SlNRC1 NBD-HD1-WHD (PDB code: 6S2P, https://www.rcsb.org/structure/6S2P) and the SlNRC1-SS15 complex (PDB code: 8BV0, https://www.rcsb.org/structure/8BV0) for alignment are obtained from PDB. The sequence of SlNRC2 is available in the Sol Genomics Network (SGN) database under accession number Solyc10g047320, and sequences of Rx and CP are available at GenBank under accession codes CAB50786 and CAA84016. Full version of gels and blots are provided in Supplementary Information Fig. 1. Primers used in this study are provided in Supplementary Table S1. Original data points in graphs are shown in the Source Data files.

Research involving human participants, their data, or biological material

Policy information about studies with human participants or human data. See also policy information about sex, gender (identity/presentation), and sexual orientation and race, ethnicity and racism.

Reporting on sex and gender	N/A
Reporting on race, ethnicity, or other socially relevant groupings	N/A
Population characteristics	N/A
Recruitment	N/A
Ethics oversight	N/A

Note that full information on the approval of the study protocol must also be provided in the manuscript.

Field-specific reporting

Please select the one below that is the best fit for your research. If you are not sure, read the appropriate sections before making your selection.

☒ Life sciences ☐ Behavioural & social sciences ☐ Ecological, evolutionary & environmental sciences

For a reference copy of the document with all sections, see nature.com/documents/nr-reporting-summary-flat.pdf

Life sciences study design

All studies must disclose on these points even when the disclosure is negative.

Sample size	The N.benthamiana infiltration experiments consisted of a total of six to ten replicates. Six to ten replicates of Hypersensitive response experiment were deemed sufficient due to any variation of protein expression in the leaves. The Ion leakage assay consisted of three independent experiments and each experiment contained five biological replicates. Total fifteen replicates were deemed sufficient due to the consistency of the results.
Data exclusions	No data were excluded from the analyses.
Replication	The purification experiment of SlNRC2 dimer and tetramer, and filament proteins were performed more than 3 times. For the experiment of cell death phenotypes mediated by SlNRC2 oligomer-disrupting mutations, SlNRC2-eGFP variants, Rx-HA-StrepII were co-expressed with or without CP-FLAG in nrc2/3/4 mutant leaves were performed for 6 or 10 replicates, respectively. For the experiment of Hypersensitive response phenotypes of SlNRC2 and SlNRC2 IP binding mutants in Rx triggered cell death. Co-expression of SlNRC2 or SlNRC2-eGFP variants, Rx-HA-StrepII and the CP-FLAG in N. benthamiana nrc2/3/4 mutant leaves, which were performed at least 8 replicates. CoIP assay for testing self-association of SlNRC2 oligomer-disrupting mutations were performed twice with similar results. Ion leakage assay of SlNRC2 oligomer-disrupting mutations in Rx triggered cell death were performed 3 times (n = 15, 5 biological independent samples for each experiment) independently. Ion leakage assay of SlNRC2 variants of IP6/5 binding sites in nrc2/3/4 mutant N. benthamiana leaves were performed 3 times(n = 15, 5 biological independent samples for each experiment) independently. qRT-PCR assay to quantify PVX virus load were performed 3 times independently. Blue Native-PAGE assay testing the oligomeric status of SlNRC2 and its variants were performed 3 times independently with similar results.

Randomization	Plant material was selected randomly from a given batch and analyzed equally. Randomization was deemed unnecessary as no sub-sampling was done.
Blinding	Blinding was not relevant to our study as we performed experiments in plant and insect cells, hence it does not include clinic trials. In plant and insect cell experiments, biology blinded or double-blinded studies are uncommon.

Reporting for specific materials, systems and methods

We require information from authors about some types of materials, experimental systems and methods used in many studies. Here, indicate whether each material, system or method listed is relevant to your study. If you are not sure if a list item applies to your research, read the appropriate section before selecting a response.

Materials & experimental systems

n/a	Involved in the study
☐	☒ Antibodies
☐	☒ Eukaryotic cell lines
☒	☐ Palaeontology and archaeology
☒	☐ Animals and other organisms
☒	☐ Clinical data
☒	☐ Dual use research of concern
☐	☒ Plants

Methods

n/a	Involved in the study
☒	☐ ChIP-seq
☒	☐ Flow cytometry
☒	☐ MRI-based neuroimaging

Antibodies

Antibodies used	Living Colors EGFP Monoclonal Antibody (Takara, Catalog: 632569).<br>Monoclonal ANTI-FLAG M2 antibody produced in mouse(Sigma, Catalog: F1804)<br>Anti HA Antibody Rabbit mAb (Roche, Catolog: C29F4).
Validation	For iving Colors EGFP Monoclonal Antibody, the manufacture Takara declared that the quality and performance of this lot of Living Colors EGFP Antibody was tested by Western blot analysis using lysate made from a HEK 293 cell line stably expressing Aequorea coerulescens GFP. A band of approximately 30 kDa corresponding to AcGFP1 was observed in the lane oaded with the AcGFP1 cell lysate. A band of this size was not detected in the lysate of untransfected HEK 293 cells<br>For ANTI-FLAG M2 antibody, the manufacture SIGMA states that the antibody detects a single band of protein on a Western Blot from mammalian crude cell lysates and detects 2 ng of FLAG-BAP fusion protein by Dot Blot Chemiluminescent Detection.<br>In addition, antibodies were validated by the use of an empty vector negative control (to control unspeccific binding of antibodies to tobacco/agrobacterium proteins).

Eukaryotic cell lines

Policy information about cell lines and Sex and Gender in Research

Cell line source(s)	Sf21 insect cell line (Invitrogen, Catalog: 11497013)
Authentication	none of the cell lines were authenticated
Mycoplasma contamination	cell line was not tested for mycoplasma contamination.
Commonly misidentified lines<br>(See ICLAC register)	no commonly misidentified cell lines were used in this study

Dual use research of concern

Policy information about dual use research of concern

Hazards

Could the accidental, deliberate or reckless misuse of agents or technologies generated in the work, or the application of information presented in the manuscript, pose a threat to:

No	Yes	
☒	☐	Public health
☒	☐	National security
☒	☐	Crops and/or livestock
☒	☐	Ecosystems
☒	☐	Any other significant area

Experiments of concern

Does the work involve any of these experiments of concern:

No	Yes	
☒	☐	Demonstrate how to render a vaccine ineffective
☒	☐	Confer resistance to therapeutically useful antibiotics or antiviral agents
☒	☐	Enhance the virulence of a pathogen or render a nonpathogen virulent
☒	☐	Increase transmissibility of a pathogen
☒	☐	Alter the host range of a pathogen
☒	☐	Enable evasion of diagnostic/detection modalities
☒	☐	Enable the weaponization of a biological agent or toxin
☒	☐	Any other potentially harmful combination of experiments and agents

Plants

Seed stocks	Nicotiana benthamiana commonly used in plant laboratories
Novel plant genotypes	The nrc2/3/4 knock-out mutant N.benthamiana was generated by CRISPR/CAS9 system.
Authentication	The transgenic plant's Genome DNA was extracted and genotyped using PCR amplification with the respective primers. The amplified DNA fragments were sequenced and compared to the sequence of wild type N. benthamiana. In T2 transgenic lines, isolate the NRCs knock out mutants by choosing the plants that without HR after infiltration of Rx and CP elicitor.
